# Polarize the Solvent to Regulate the Intermediate Phase and Dynamic Crystallization of Perovskite Films

**DOI:** 10.1002/adma.202519793

**Published:** 2026-01-13

**Authors:** Zhuoqiong Zhang, Yunfan Wang, Weizhen Wang, Yulan Huang, Shanchao Ouyang, Yonggui Sun, Fei Wang, Xianfang Zhou, Guichuan Xing, Shu Kong So, Guozhong Xing, Hanlin Hu, Songhua Cai, Sai‐Wing Tsang, Tom Wu

**Affiliations:** ^1^ Department of Applied Physics The Hong Kong Polytechnic University Hong Kong SAR P. R. China; ^2^ Department of Materials Science and Engineering City University of Hong Kong Hong Kong SAR P. R. China; ^3^ Hoffmann Institute of Advanced Materials Shenzhen Polytechnic University Shenzhen P. R. China; ^4^ Joint Key Laboratory of the Ministry of Education Institute of Applied Physics and Materials Engineering University of Macau Macau P. R. China; ^5^ Department of Physics and Institute of Advanced Materials Hong Kong Baptist University Hong Kong SAR P. R. China; ^6^ Key Laboratory of Microelectronics Devices & Integration Technology Institute of Microelectronics Chinese Academy of Sciences Beijing P. R. China

**Keywords:** crystallization dynamics, intermediate phase, perovskite solar cells, polar polymer, solvent engineering

## Abstract

Perovskite solar cells (PSCs) have demonstrated substantial potential due to their superior optoelectronic performance, but rapid and often poorly controlled crystallization during dynamic solution processing frequently leads to defective crystal growth and compromised film quality. Herein, we introduce a strategy utilizing polar polymers to intricately regulate solvent polarity and evaporation kinetics, thereby modulating the dynamics of perovskite crystallization. Particularly, the strongly polarized, high‐population fluorinated groups in poly(pentafluorostyrene) strongly interact with solvent molecules in the precursor solution, stabilizing the solvent‐containing intermediate phase and controlling the exfoliation of solvent molecules during perovskite crystallization. Direct imaging by scanning transmission electron microscopy reveals the structure of the intermediate phase, and in situ optical studies demonstrate that the regulated crystallization suppresses defect formation and improves film quality. Consequently, inverted PSCs fabricated via this new solvent engineering strategy achieve an efficiency of 26.4% and retain 92% after 1000 h of continuous illumination, underscoring the effectiveness of this strategy of polarizing the solvent.

## Graphical abstract

A solvent engineering strategy using polar polymers is introduced to regulate perovskite crystallization dynamics in solar cells. Strongly polarized fluorinated groups stabilize the intermediate phase and control solvent exfoliation, leading to high‐quality films with suppressed defects. This approach enables inverted perovskite solar cells to achieve 26.4% efficiency and excellent long‐term stability under continuous illumination.

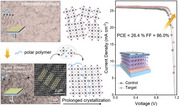

## 1 Introduction

Organic‐inorganic metal halide perovskite solar cells (PSCs) have garnered substantial attention over the past decade due to their high‐power conversion efficiencies (PCEs), cost‐effectiveness, and potential for scalable production [1, 2]. Remarkably, their PCEs have now reached over 27%, rivalling those of commercial silicon solar cells and positioning PSCs as promising candidates for next‐generation photovoltaic technologies [3]. To date, high‐efficiency PSCs are typically fabricated based on the antisolvent‐assisted one‐step solution process, a simple and efficient method for producing dense and high‐crystallinity perovskite thin films [4, 5]. Xiao et al. pioneered the use of antisolvent to accelerate solvent extraction during the spin‐coating process, achieving fast nucleation and forming uniform and full‐coverage perovskite films [6]. However, the process also caused rapid and uncontrollable crystallization after the antisolvent was dripped, which resulted in small and non‐homogeneous grain sizes. This side effect can increase the defect densities and negatively impact the device performance. To address this issue, Jeon et al. prepared methylammonium lead halide (MAPbX_3_) precursor solution by mixing approximately 43% dimethyl sulfoxide (DMSO) into a γ‐butyrolactone solvent [7]. Upon dripping the antisolvent, the residual DMSO can coordinate with lead iodide (PbI_2_) and methylammonium iodide (MAI) to form a Lewis acid‐base adduct. The MAI‐DMSO‐PbI_2_ adduct, or the so‐called intermediate phase, can impact the crystal growth pathway by slowing down crystallization, favoring the growth of continuous and dense perovskite films. This innovation improved the PCE while suppressing the hysteresis and established the one‐step method as a reliable and widely adopted technique to prepare perovskite films. However, the microstructure of this intermediate phase was not elucidated, and the lack of knowledge on the crystallization dynamics hampers the optimization of the solution processing of perovskite films.

In recent years, formamidinium (FA)‐based perovskite has increasingly replaced conventional MA‐based perovskites in the development of high‐efficiency single‐junction PSCs, owing to its optimal bandgap (1.48 eV) and superior thermal stability [8, 9, 10, 11, 12]. In FA‐based mixed‐cation PSCs, MA‐containing additives are often employed to achieve record‐high PCE values [13, 14, 15, 16]. Pure FAPbI_3_‐based perovskite films do not exhibit any detectable intermediate phase because the coordination between FA and DMSO is significantly weaker than that between MA and DMSO [17]. In mixed‐cation FAMA‐based perovskites, the reduced formation of the MAI‐DMSO‐PbI_2_ intermediate phase compromises the nucleation and crystallization process, presenting challenges for achieving high‐quality perovskite films [18]. In 2018, Lee et al. found that the weak hydrogen bond between DMSO and FA was the primary cause of the unstable FAI‐DMSO‐PbI_2_ adduct [19]. Hence, the team proposed a solvent‐engineering approach of replacing DMSO with *N*‐methyl‐2‐pyrrolidone (NMP), a Lewis base with stronger hydrogen bonding ability, facilitating the formation of a stable FAI‐NMP‐PbI_2_ adduct in the perovskite precursor solution. This approach improved the PCE of FA‐based PSCs to over 20% (stabilized PCE of 19.34%). Similarly, Wang et al. used a strong coordinating solvent, 1,3‐dimethyl‐3,4,5,6‐tetrahydro‐2(1H)‐pyrimidinone (DMPU), to form a stable solvent‐adduct with the perovskite precursor even at high ambient temperatures, widening the processing window of high‐efficiency PSCs [20]. While selecting different solvents has proven effective in stabilizing intermediate phases and improving film quality, this strategy suffers from some shortcomings. For example, excessive accumulation of intermediate phases can delay crystallization and produce an uneven grain distribution. Furthermore, these adducts are thermodynamically stable and often require temperatures exceeding standard annealing conditions to decompose; consequently, detrimental residues persist, compromising the long‐term stability of PSCs [21, 22]. Therefore, additives such as MACl are often employed to facilitate the formation of solvent‐adduct intermediate phases, improving film quality, notably by increasing the grain size in the final film [23]. However, during annealing, the volatility of such additives can cause non‐uniform films and recombination losses, limiting their effectiveness [24, 25]. These challenges underscore the need for new solvent‐engineering approaches that must be cost‐effective, reliable, and highly tunable. It has become a consensus that intermediate phases may be beneficial and offer precise control of the perovskite crystallization, but they must be fully converted to the perovskite structure without altering the physical properties of the final films [26, 27]. Addressing these challenges remains vital for advancing the scalability and commercial viability of PSCs.

In this work, we present a new solvent engineering strategy using a series of polar polymers to regulate intermediate phases and crystallization dynamics in perovskite films. Specifically, poly(pentafluorostyrene) (PPFS), with its highly polarized fluorinated groups, is employed to enhance the DMSO‐containing intermediate phase. The precisely controlled evaporation of solvent facilitates the retardation of crystallization while circumventing the risk of solvent trapping, facilitating the high‐quality crystallization of the perovskite films. The effectiveness of this strategy was directly demonstrated using a suite of techniques, including low‐dose scanning transmission electron microscopy (STEM), in situ X‐ray diffraction (XRD), and in situ optical techniques. STEM, in particular, provided the direct atomic‐scale visualization of the microstructure of the intermediate phase and its interface with the perovskite structure. As a result of the highly controlled crystallization of perovskite films, the PPFS‐tailored cells exhibit a remarkable PCE of 26.4% with an impressive fill factor (FF) of 86.0%. Under steady‐state light illumination and a constant bias voltage corresponding to the maximum power point (MPP) of the initial state, the device maintained 92% of its original power output after operating continuously for 1000 h. The comprehensive characterization data indicate that polarizing the solvent using judiciously chosen polymer is a viable approach to regulate the critical crystallization process of perovskite films without bringing about any negative effect. This approach, based on polar polymers, has the potential to be generalized to other solution‐processed materials involving intermediate phases, offering a reliable pathway toward high‐performance optoelectronics with improved stability and scalability.

## 2 Results and Discussion

### 2.1 Polar‐Polymer‐Enabled Solvent Engineering

**FIGURE 1:**
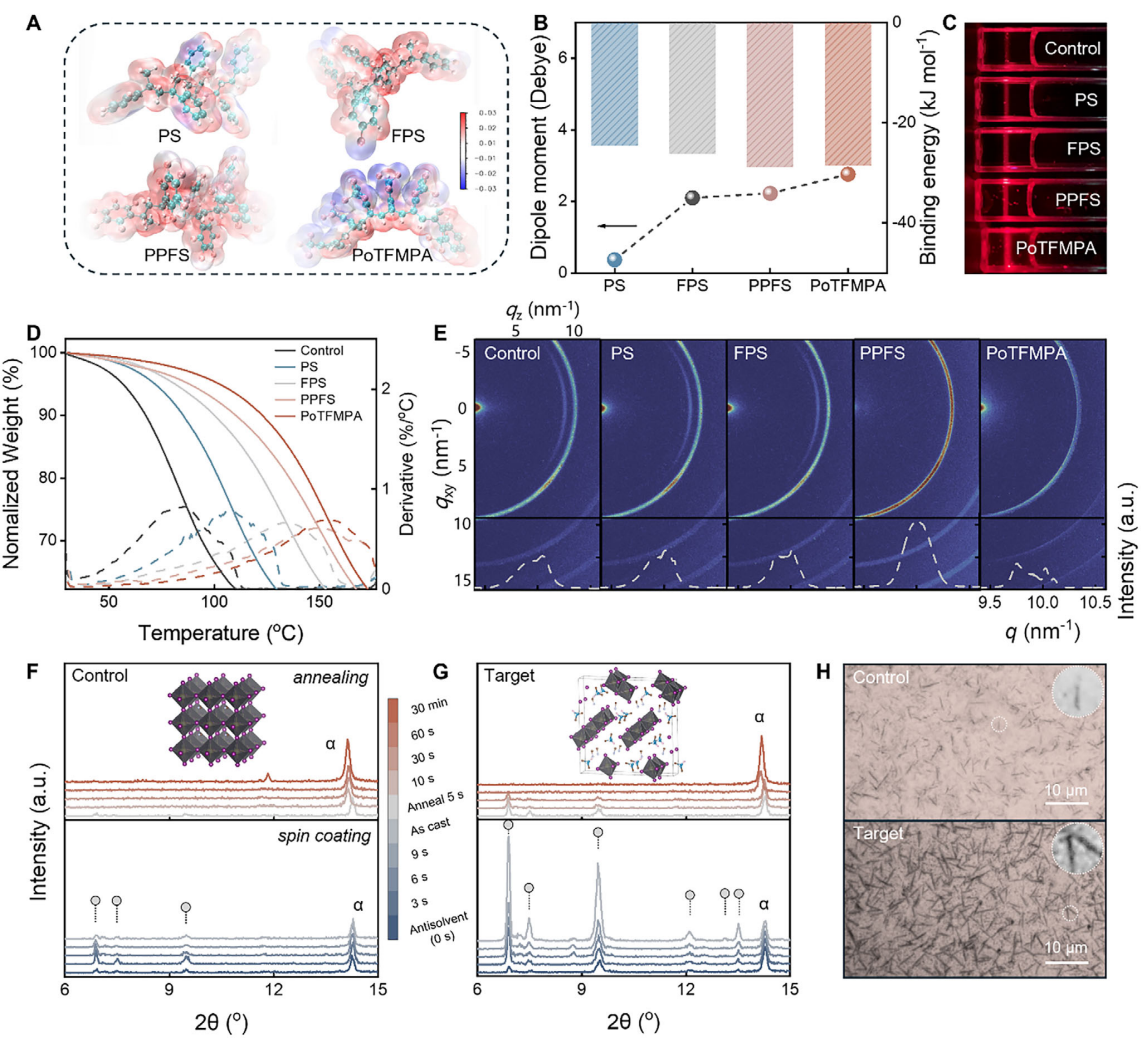
(A) Surface electrostatic potential distribution of four polymer fragments. In the illustrations of molecules, the blue, red, and white balls represent carbon, fluorine, and hydrogen atoms, respectively. (B) Dipole moments of monomers and their corresponding binding energies with DMSO. (C) Tyndall effect measurements of the control and polymer‐incorporated perovskite precursor solutions. The photo was rotated clockwise by 90°. (D) Thermogravimetric analysis (TGA) curves and their derivatives (dashed lines) of the solutions. (E) GIWAXS patterns of control and polymer‐optimized perovskite films. Red regions highlight strong diffraction signals for specific crystal planes with preferred orientation, while light blue regions indicate weaker diffraction intensity for corresponding planes. Below: Integrated 2D GIWAXS patterns of the perovskite films. In situ tracking of X‐ray diffraction of the (F) control and (G) target perovskite films. The blue and red curves represent data taken during the spin‐coating and annealing processes, respectively. Circles denote the diffraction peaks from the intermediate phase. (H) Optical images of the wet perovskite films, highlighting the 1D morphology of the intermediate phase crystallites.

We hypothesize that the asymmetric charge distribution and the associated dipole moment of polar polymers can regulate the nucleation and crystallization processes of solution‐processed perovskite films, leveraging the electrostatic interaction between the polymer dipoles and the precursor solvent. Compared with small molecules, polymers offer a much higher density of polar moieties with a rich configuration and dipolar diversity. Figure 1A shows the calculated surface electrostatic potential distributions for four polymers selected in the current case study, including the common polystyrene (PS) and its fluorinated functional group derivatives poly(4‐fluorostyrene) (FPS), PPFS, and poly(2‐trifluoromethylphenyl acetylene) (PoTFMPA), each with an increasing number of fluorinated groups. The corresponding monomer dipole moments are calculated as 0.37, 2.10, 2.22, and 2.76 Debye, respectively, as shown in Figure 1B (details can be found in the experimental section). The fluorinated groups can effectively enhance the dipole moments, which is expected to strengthen the interaction of the polar polymers with the solvent molecules. The calculated interaction energies reflect the strength of interactions between the monomer molecules and the solvent molecules [28, 29]. Mixtures of DMF and DMSO are the prevalent co‐solvents for preparing perovskite films because this combination can optimize the film formation process and the quality of the final perovskite layer. It should be noted that DMF in the mixed solvent is largely removed during the spin‐coating process due to its fast evaporation and antisolvent extraction, resulting in only DMSO remaining in the wet film. Hence, only the binding energies between the polymer monomers and DMSO were considered. As shown in Figure 1B, the monotonously increasing trend supports the enhanced polymer‐solvent interactions by fluorinated groups. Our results imply that the dipole moment of the monomer is an excellent descriptor for its interactions with the solvent molecules.

To further experimentally investigate the interactions, we performed Fourier Transform Infrared Spectroscopy (FTIR) to examine the effect of the polymer on the S = O stretching vibration in DMSO, which is located at approximately 1041 cm^−1^ (Figure S1) [30]. The addition of polymers causes a shift to lower wavenumbers, with the shift being more pronounced for polymers with higher polarity, indicating stronger interactions between the polymers and DMSO. To investigate how polymer influences solvent characteristics, we measured the solvent polarity parameter, commonly denoted as *E*_T_(30), using the solvatochromism of the dye betaine [31]. As shown in Figure S2, the absorption spectrum of DMSO solvent exhibits a systematic blueshift upon the incorporation of various polymers. Specifically, the normalized polarity parameter, *E*_T_^N^(30), converted into SI units using polar and nonpolar reference solvents, increased from 0.44 for pure DMSO to a range of 0.45–0.47 in the presence of the polymer investigated. This increase in solvent polarity may strengthen interactions with perovskite precursors through solvent‐involved intermediates [32]. The effective modification of the perovskite precursor solutions by the polar polymer was further confirmed by the measurements of the Tyndall effect, which originates from light‐colloid scattering (Figure 1C) [9]. As the polymer polarity increases, stronger light scattering is observed, which is due to the formation of a large‐size polymer‐PbX_2_‐DMSO host‐guest complex, reflecting the strong interaction between the polymer and solvent [9, 33].

As shown in the thermogravimetric analysis (TGA) (Figure 1D), the polymer‐tempered perovskite precursor solutions exhibit much slower continuous evaporation due to the coordination of the polymer with the solvent. This suppression of volatility is also observed in pure DMSO containing the polymer (Figure S3). This effect is further illustrated in Figure S4, which captures the evaporation process of the solutions. Basically, the fluorinated functional groups of the polar polymers “grasp” the solvent molecules, preventing the vapor generation, and the alteration of this key solvent property stabilizes the precursor solution, remarkably extending the lifetime of the solvent‐adduct intermediate phase in the wet films during the solution processing. In addition, it is not a surprise that the viscosity of the solution, modified by the polymer incorporation, significantly increases from 0.7 × 10^−2^ to 2.6 × 10^−2^ Pa·s at a shear rate of 1 s^−1^, as revealed by the rheology measurement (Figure S5). This change can influence the diffusion of perovskite ions in the wet film, thereby regulating the subsequent crystallization process [34]. Generally, solvent diffusion and evaporation are critical factors that influence grain nucleation and crystal growth in perovskite films. Consequently, we anticipate that perovskite films prepared using the polarity‐tailored solvent will exhibit higher film quality since this strategy provides an additional degree of freedom in the optimization of solution processing. Furthermore, we should point out that no solvent residue was detected after the annealing treatment, warranting the synthesis of dry perovskite films with complete solvent evaporation (Figure S6).

To investigate the effect of polymers with different polarities on the perovskite crystallization, grazing incidence wide‐angle X‐ray scattering (GIWAXS) was performed. Figure 1E presents the 2D GIWAXS patterns of the control perovskite film as well as those modified with PS, FPS, PPFS, and PoTFMPA polymers. The polymers were introduced via the antisolvent at the optimized concentration of 0.5 mg mL^−1^. The diffraction ring located at 9 nm^−1^ is assigned to PbI_2_. It is noted that the peak intensity of PbI_2_ is attenuated in the perovskite films with PPFS and PoTFMPA treatments. The bright ring at *q* = 10 nm^−1^ is assigned to (100) perovskite crystal plane, with the radial integration results also shown. The intensity of the (100) peak is significantly enhanced after the PPFS polymer treatment, indicating the highest crystallinity of the modified perovskite film. Therefore, in the following experiments, PPFS was selected as a modifier to optimize the quality of perovskite films, and samples treated without and with PPFS treatment are labelled as “control” and “target”, respectively.

To better understand how the polymer influences the perovskite crystallization, we conducted in situ XRD tracking of the perovskite film fabrication process, during both the spin coating and annealing stages. As shown in Figure 1F,G, upon antisolvent dripping, a solvent‐adduct intermediate phase (2*θ* = 6.73°, 7.38°, and 9.33°) is formed in the film, which is related to the MAI‐PbI_2_‐DMSO phase and matches well with the XRD patterns simulated from the single‐crystal structure (Figures S7 and S8) [35]. This intermediate phase has been reported in PSCs, even in the presence of only a small amount of MA^+^ in the perovskite precursor solution [24, 36]. During the subsequent spin‐coating and annealing processes, the intermediate phase gradually transforms into the α‐phase of perovskite, without any observable formation of the δ‐phase. Notably, the peak of the α‐phase perovskite gradually shifts to low angles due to the progressive replacement of Cl ions by larger I ions [37]. In comparison to the control sample, the peaks of the intermediate phase in the target film are much stronger and more stable, persisting even after 30 s of annealing. This behavior may be attributed to the retarded solvent exfoliation caused by the dipole interaction with the polymers, as supported by the presence of more residual solvent in the target wet film before the annealing treatment (Figure S9).

Furthermore, larger needle‐shaped intermediate crystals with a higher density appear in the target film compared with the control one, as evidenced by optical, scanning electron microscope (SEM), and atomic force microscopy (AFM) images of the as‐cast films (Figure 1H; Figures S10 and S11). The needle shape can be attributed to the ribbon‐like motif of face‐sharing lead iodide octahedra in the intermediate phase, which has been reported previously in the literature [22]. A similar trend is obtained when the polymer was directly added to the DMSO precursor solution containing only MAI and PbI_2_ (Figures S8, S12–S15), indicating that the polymer's effect stems from direct interactions with these core components (MAI, PbI_2_, DMSO) and is independent of other additives. It can be concluded that the polymer incorporation in the solvent facilitates the formation and growth of the intermediate phase. This effect is significantly influenced by the strong polymer–solvent interactions rather than arising from direct polymer–precursor coordination, as evidenced by the spectroscopic data shown in Figure S16. The stable intermediate phase, in turn, can gradually release the precursor ions, which, as we will see, significantly impacts the nucleation and crystallization processes.

### 2.2 Direct Imaging of the Intermediate Phase with STEM

**FIGURE 2:**
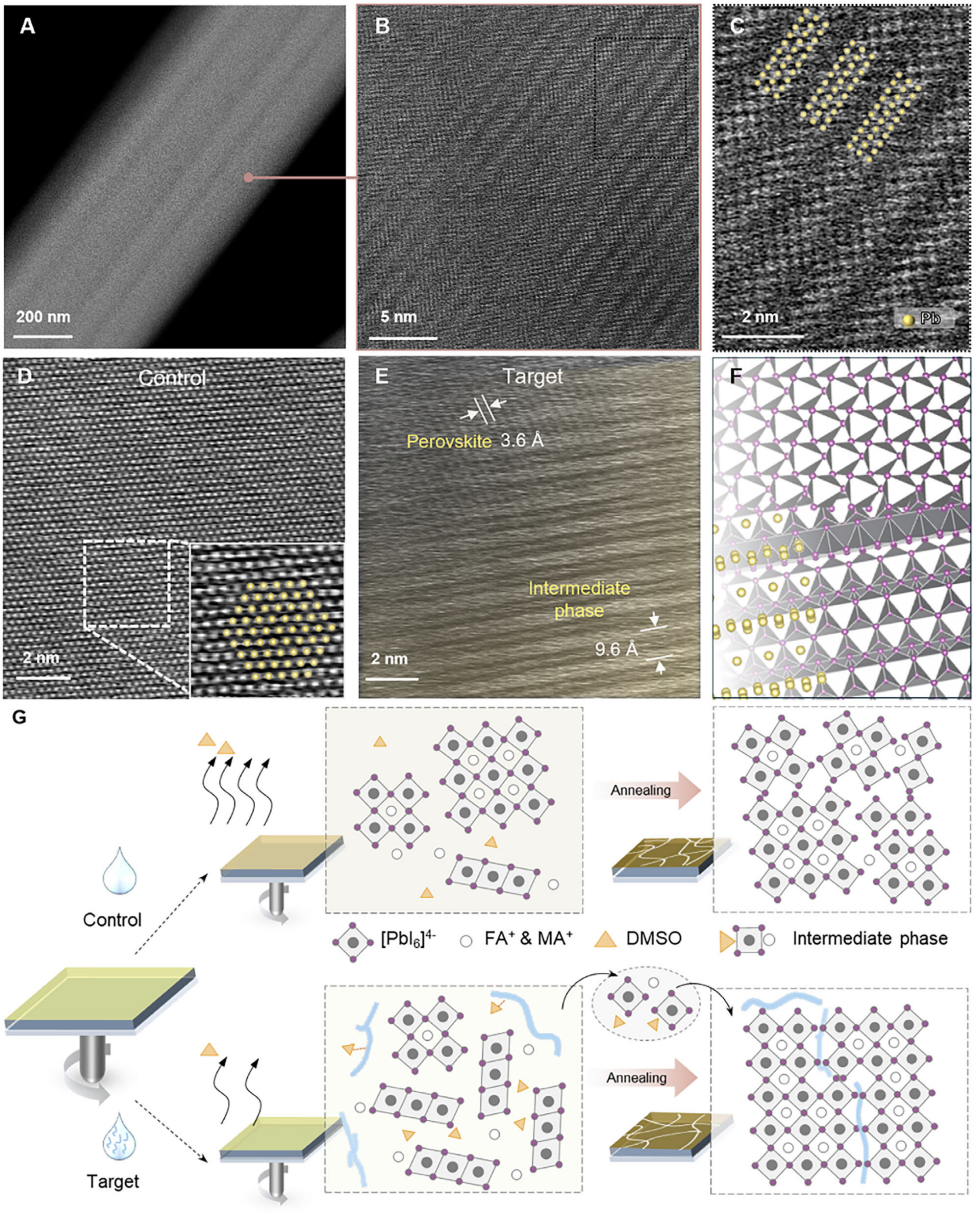
(A) Low‐resolution STEM image of the PPFS‐modified target sample. (B) High‐resolution HAADF‐STEM image of the target sample. (C) Enlarged view of the square region in (B). (D) HAADF‐STEM image of MAI‐PbI_2_‐DMSO after annealing for 2 min, with the inset revealing the perovskite atomic structure, overlaid with the standard structure model. (E) HAADF‐STEM image of the target sample after annealing for 2 min, showing the coexistence of the perovskite and the intermediate (highlighted in yellow) phases. (F) Corresponding atomic structure model: top for the perovskite phase and bottom for the intermediate phase. The yellow and purple spheres represent lead and iodine atoms, respectively. (G) Schematic illustration of the perovskite crystallization pathways: without polymer, rapid crystallization produces uneven‐sized grains with defective boundaries, while polymer addition stabilizes the intermediate phase, enabling the slow growth of high‐quality perovskite films.

To reveal the critical role of the intermediate phase in the perovskite processing, we then carried out low‐dose STEM imaging to take advantage of its exceptional spatial resolution. While STEM has been extensively utilized for characterizing metal halide perovskite recently, its application to characterizing intermediate phases has been limited due to their metastable nature and their presumed instability under the radiation of even a moderate electron dose [38]. In our STEM characterizations, the electron probe beam current was minimized to 1 pA, and the average dose rate was reduced to 6 eÅ^−2^ s^−1^ to ensure that the intrinsic structures of the intermediate and perovskite phases remained undamaged during the experiment [39]. Low‐magnification high‐angle annular dark‐field (HAADF)‐STEM images clearly illustrate the characteristic needle‐like morphology of the MAI‐PbI_2_‐DMSO intermediate phase structure in both control and target samples (Figure S17; Figure 2A), aligning with observations from optical and SEM images. Further examination using low‐dose, high‐resolution HAADF‐STEM imaging (Figure S18; Figure 2B) reveals a pronounced 1D structural motif. The atomic column arrangements in both the control and target samples closely match the proposed structure model, suggesting that the incorporation of polymer does not significantly alter the overall structure. This result was confirmed through imaging multiple samples, demonstrating phase purity and spatial homogeneity (Figure S19). Additionally, selected area electron diffraction patterns further confirm that the sample with polymer treatment retains its structural integrity without decomposition or phase transformation (Figure S20). From extensive imaging of many local areas, we found that the addition of polymer appears to enhance the structural continuity, likely through improving the overall sample crystallinity. Comprehensive analysis of the real‐space images, complemented by the corresponding fast Fourier transform patterns, confirms that the intermediate phase has an orthorhombic structure with the composition of MA_2_Pb_3_I_8_·2DMSO (space group *Cmc2*_1_) (projected along the [010] crystallographic direction in the particular case shown in Figure S21) [35]. The periodic variation in the tilt angle of Pb atoms further corroborates the proposed structural model, as depicted in Figure S22 and Figure 2C.

Upon brief heating at 100°C for 2 mins, atomic‐resolution imaging reveals that the intermediate phase undergoes the phase transition into the perovskite phase. As shown in Figure 2D, the MAI‐PbI_2_‐DMSO intermediate is fully transformed into the perovskite phase. However, in the PPFS‐modified target sample (Figure 2E), two distinct lattice patterns were observed after the same annealing procedure: the stripe‐type structure with a periodic spacing of 9.6 Å corresponds to the 1D intermediate phase (highlighted in yellow), and the corresponding Fast Fourier transform pattern is shown in Figure S23, while the 3.6 Å stripe structure is identified as the tetragonal perovskite structure viewed along the [011] crystallographic direction. These two phases are well‐integrated at the microscopic scale (Figure 2F) and separated by a gradual interface.

These real‐space imaging results provide direct evidence supporting the widespread hypothesis that the intermediate phase undergoes a thermally activated transformation into the photo‐active perovskite phase. Based on the results above, we illustrate the effect of polar polymer on the crystallization process of perovskite films in Figure 2G. In the absence of the polymer, the perovskite film crystallizes rapidly, resulting in smaller grains with defective boundaries. By contrast, introducing the polymer into the precursor solution via antisolvent slows down the exfoliation of residual DMSO in the wet film, stabilizing the intermediate phase. The regulated solvent dynamics and the intermediate phase crystallization, in turn, enable a controlled release of precursor ions, thereby mitigating the excessively rapid crystal growth, and as a result, high‐quality perovskite films with larger crystals and fewer defects are obtained.

### 2.3 In Situ Observation of the Crystallization Kinetics

**FIGURE 3:**
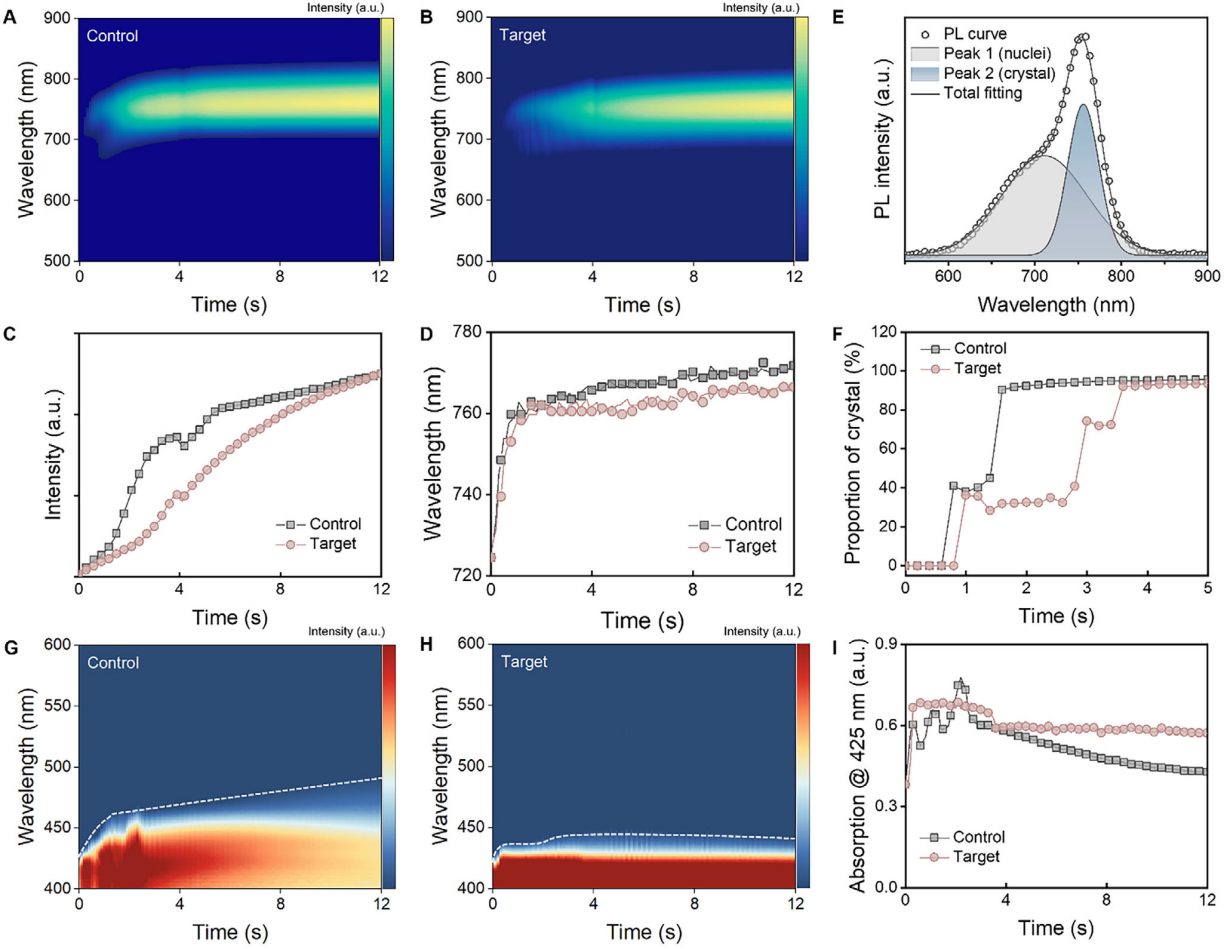
2D contour maps of PL spectra during spin coating for perovskite films without (A) and with (B) polymer treatment. Corresponding PL intensity (C) and peak position (D) trends extracted from the contour maps. (E) PL signal fitted with two Gaussian peaks. (F) Evolution of crystal phase proportions during spin coating. 2D contour maps of UV–vis spectra during spin coating for perovskite films without (G) and with (H) polymer treatment. (I) Extracted absorption intensity evolution at 425 nm.

To investigate the real‐time impact of polar polymer on perovskite crystallization kinetics, we employed in situ optical techniques to monitor the sample properties during the spin‐coating and annealing processes. The experimental setup for in situ photoluminescence (PL) and UV–vis absorption measurements is illustrated in Figures S24 and S25, with detailed procedures provided in the experimental section. Figure 3A,B presents 2D contour plots of the in situ PL data obtained from the spin‐coating process. At the point of “0 s”, which marks the moment of the antisolvent being dripped, nucleation initiates and produces detectable PL signals, particularly in the control sample. In contrast, the polymer‐modified sample exhibits a weaker initial PL response, indicative of slower formation of luminous nuclei. To further compare nucleation behavior, Figure 3C,D show the extracted trends of PL intensity and peak position from the contour maps. In the control film, PL intensity rises rapidly, accompanied by a pronounced redshift to 770 nm. This shift signifies a diminishing quantum confinement effect, indicative of perovskite grain growth [40, 41]. In contrast, the target sample exhibits a slower PL intensity increase and a gradual redshift to 765 nm, indicating retarded nucleation and hindered crystal growth [42]. This may be attributed to the presence of more strongly bonded intermediate phases (non‐luminescent) in the target film mentioned earlier. The asymmetrical shape of the early‐stage PL spectra reflects a coexistence of perovskite nuclei and bulk crystal phases emitting at distinct wavelengths [20]. To resolve this, the spectra were deconvoluted using a two‐Gaussian fitting model (Figure 3E and raw data in Figure S26), identifying peaks at 760 nm (bulk perovskite phase) and shorter wavelengths (growing nuclei) [42, 43]. By integrating the intensity of these fitted peaks, we quantified the temporal evolution of the crystal phase proportion during spin coating (Figure 3F). In the control sample, the perovskite crystal fraction surged from 0% to 95% within 1.5 s after antisolvent dripping. In contrast, the polymer‐modified film reached only 30% at 3 s and gradually approached 95% by 4 s. These results suggest that the polymer additive stabilizes intermediate phases, slows large‐crystal formation, and promotes a more complete and controlled nucleation process.

To gain further insight into crystallization dynamics, in situ UV–vis absorption spectra were acquired during spin coating, providing additional insights into non‐luminescent material evolution. In the control sample (Figure 3G), the absorption edge shifts steeply to longer wavelengths (∼500 nm), signaling more rapid perovskite crystal growth. By contrast, the polymer‐modified sample (Figure 3H) retains characteristic absorptions of intermediate phases and small‐size nuclei (∼425 nm) for an extended duration [44]. The absorption evolution at 425 nm was tracked to monitor the phase transition to the perovskite phase [45]. In the control film, absorption at 425 nm increased immediately following antisolvent dripping, followed by a rapid decrease, marking fast nucleation and transition to the perovskite phase (Figure 3I). However, the target sample showed a much slower decline in the 425 nm absorption, indicating retarded crystallization due to the stabilization of the intermediate phase. In situ monitoring during the subsequent annealing process (Figure S27) reveals a rapid decay in PL intensity for the control film, particularly within the first 30 s, indicating that thermal quenching quickly dominates [18, 42]. In contrast, the polymer‐modified film exhibited a significantly slower decline extending to 50 s. This prolonged resistance against the thermal quenching effect confirms the retarded crystallization kinetics in the polymer‐modified film. This behavior is further corroborated by the evolution of UV–vis absorption spectra during annealing (Figure S28). Collectively, the in situ PL and absorption results demonstrate that nucleation and crystallization occur more rapidly in the control sample. In comparison, the incorporation of polymer in the target sample effectively stabilizes the intermediate phase during the spin‐coating process. This stabilization slows the release of precursor materials from the intermediate phase, mitigating fast and uncontrollable crystallization and enabling a more regulated crystallization process.

### 2.4 Improved Film Quality Induced by Polymer‐Regulated Crystallization

**FIGURE 4:**
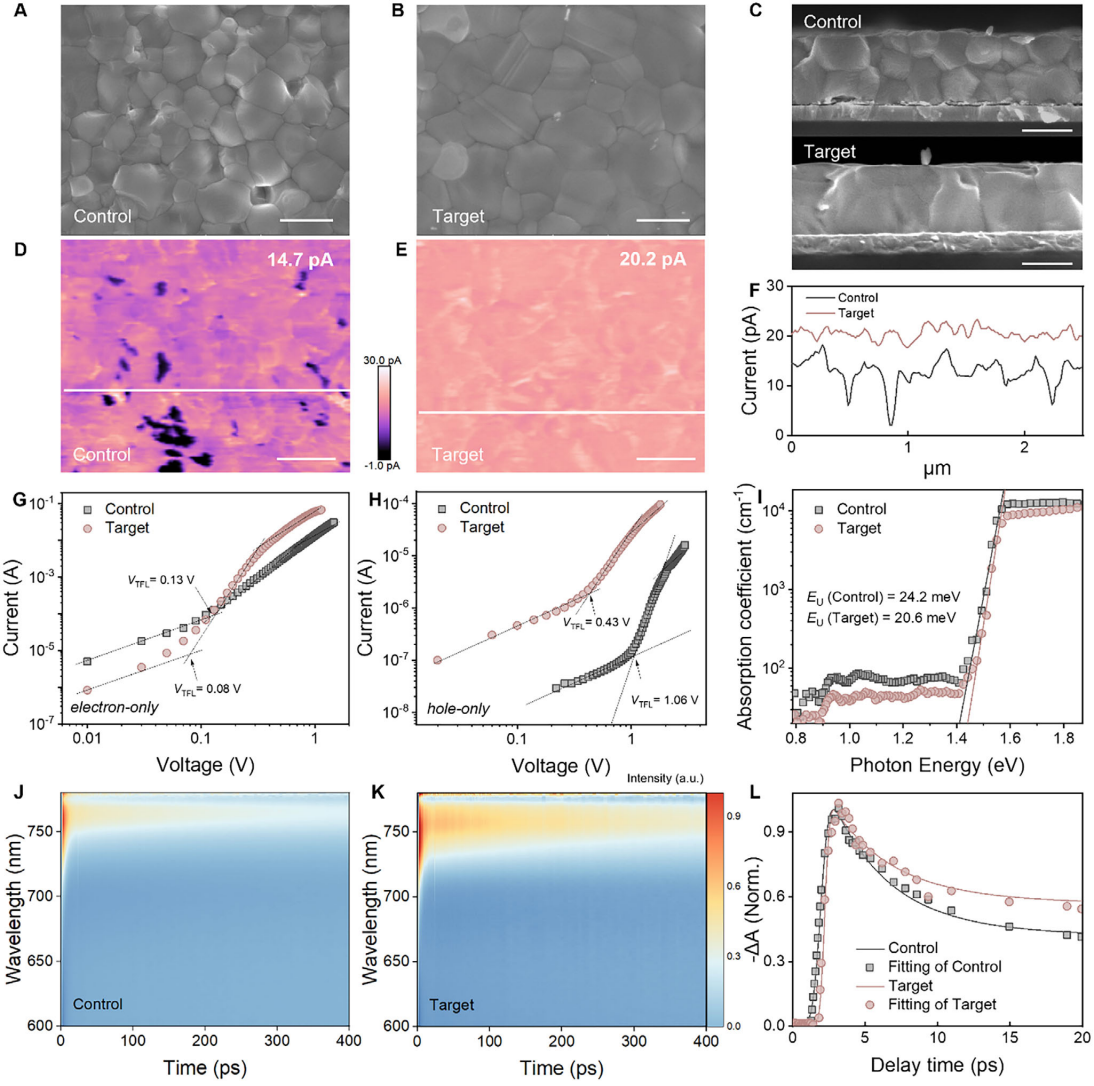
Surface SEM images of the control (A) and target (B) perovskite films, as well as (C) corresponding cross‐section SEM images, indicating the improved crystallinity after polymer modification and the absence of any observable polymer aggregation. Scale bar: 500 nm. Conductive‐AFM surface current images of (D) the control and (E) the target films. Scale bar: 500 nm. (F) Corresponding line profiles of the surface current. *J*–*V* curves of (G) electron‐only and (H) hole‐only devices under dark conditions. (I) PDS spectra of the control and target perovskite films. Pseudo‐color femtosecond transient absorption (fs‐TA) spectra of (J) the control and (K) the target perovskite films. (L) Corresponding photobleaching decay curves deduced from the fs‐TA data.

Benefiting from the modulation of the polymer on the nucleation and crystallization process of perovskite, the target film is expected to achieve higher quality. To verify this, we investigated the effects of PPFS on the perovskite film properties, focusing on morphology, energetic disorder, and optoelectronic characteristics. The film surface morphology was first examined by SEM, with the results shown in Figure 4A,B, and the cross‐sectional microstructure is shown in Figure 4C. The target perovskite film exhibits a more compact and uniform surface with enlarged perovskite grains as compared to the control film, which can be attributed to the slow crystallization induced by PPFS. Additionally, cross‐sectional images reveal that the grains in the target film are more coherent along the vertical direction, likely due to the uniform presence of the polymer, promoting fully grown grains. The lack of horizontal grain boundaries in the target film mitigates sluggish cross‐grain charge hopping and improves the transport properties of the perovskite film. In line with the SEM results, AFM characterization reveals that the polymer addition reduces the surface roughness of the perovskite from 25 to 19 nm (Figure S29), suggesting that the target film has a smoother surface, beneficial to interface contact and homogeneous charge transport.

To gain insights into the spatial distribution of polymer in the perovskite films, time‐of‐flight‐secondary‐ion mass spectrometry (ToF‐SIMS) was performed to track the polymer‐specific F signal. It was found that the polymer is uniformly distributed throughout the bulk and surface of perovskite films (Figure S30). This macroscopic spatial uniformity enables consistent control over the crystallization process across the entire film, leading to improved overall crystal quality. In addition, even if polymers may reside microscopically at the grain boundaries, the observed absence of any excessive local aggregation of the insulating polymer ensures the preservation of the intrinsic superior charge transport properties of perovskite films. Indeed, as shown in Figure 4D,E, the target film optimized with polymer gave a considerably higher and more uniform distribution of micro‐conductivity in the scanned area (20.2 pA) than that of the control one (14.7 pA), indicating reduced local resistive barriers and improved charge transport pathways at the nanoscale within the film [46]. The line profiles derived from the current mapping are presented in Figure 4F, showing that the current distribution is more uniform in the presence of the polymer, which is also consistent with the fact that the polymers are distributed homogeneously throughout the whole sample, in line with the SIMS result. Confocal PL mapping also confirms the homogeneity of the perovskite layer (Figure S31), demonstrating that the polymer improves the overall film quality and the total quantity of the polar polymer additive is well adjusted so that it does not bring any detectable property deterioration. It should be noted that further increasing the polymer content beyond the level of 1 mg mL^−1^ leads to a noticeable reduction in PL signals and degraded perovskite film properties (Figure S32).

In general, the optoelectronic properties of perovskite thin films are closely linked to the trap density of states, which is strongly influenced by the sample processing and the film quality [47]. To better understand the effect of PPFS on optoelectronic characteristics, space‐charge‐limited current (SCLC) measurements were employed to quantify the defect density (*N*_t_) in the perovskite materials. Using the formula *N*_t_ = (2ɛ_0_ɛ*_r_V*_TFL_)/(*q*L^2^), the electron trap density of the control film was calculated to be 8.3 × 10^14^ cm^−3^ [48], while the polymer‐tailored film exhibits a reduced trap density of 5.1 × 10^14^ cm^−3^ (Figure 4G). Consistent results showing suppressed trap densities are also observed in hole‐only devices (Figure 4H), with the detailed parameters shown in Table S1. Furthermore, the highly sensitive photothermal deflection spectroscopy (PDS) technique was utilized to characterize defect states in the perovskite films, as shown in Figure 4I. The Urbach energy (*E*_U_) is extracted from the slope of the absorption tail in the PDS spectra with the equation of $\alpha =\alpha_{0}\exp\left[\frac{h\nu -E_{g}}{E_{U}}\right]$, where α_0_ is the absorption coefficient at the bandgap energy *E*_g_, and *h*ν is the photon energy [49, 50]. The *E*_U_ for the control film is 24.2 meV, larger than that (20.6 meV) for the polymer‐treated one, suggesting the suppression of band edge disorder of perovskite films. There is also a noticeable decline in the sub‐bandgap absorption after the polymer incorporation (in the range of 0.8–1.4 eV). By applying the optical sum rule to the integrated excess absorption, we confirm that this reduction corresponds to suppressed trap densities in the polymer‐treated perovskite film [51].

In addition, femtosecond transient absorption (fs‐TA) spectroscopy was tested under an excitation laser pulse of 360 nm to study the exciton dynamics of perovskite films. Figure 4J,K show the 2D pseudo contour maps, with negative ground state photobleaching peaks at ∼760 nm attributed to carrier filling at the band edge [52]. Notably, the observed intensity extension in the target sample (Figure 4L; Table S2) reflects a longer carrier lifetime compared with the control one [53]. Furthermore, according to the time‐resolved PL data (Figure S33), the reduced carrier recombination due to polymer treatment is evidenced by a notable increase of the PL lifetime from 0.62 to 1.37 µs, which is assigned to the refined perovskite crystallization with suppressed trap density of states.

### 2.5 Photovoltaic Performance

**FIGURE 5:**
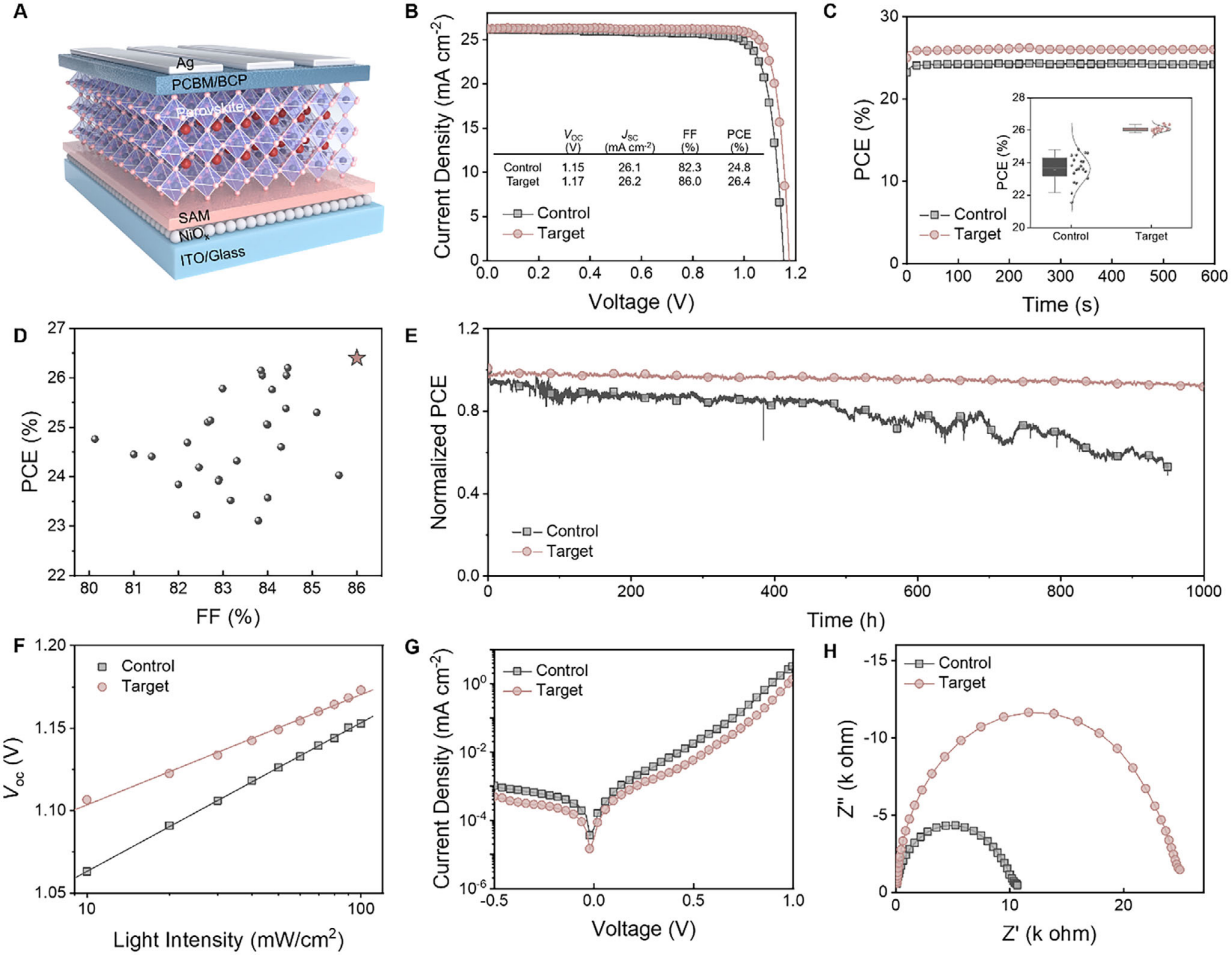
(A) Device architecture of the inverted PSC. (B) *J*–*V* curves of control and target PSCs. (C) Steady‐state power output of the devices. The inset shows the PCE distribution of the PSCs. (D) Summary of reported PCE and FF for recent PSCs with polymer‐modified active layers (star indicates our result). (E) Device stability of encapsulated devices. (F) Light intensity‐dependent VOC tests. (G) Dark *J*–*V* curves of the devices. (H) Nyquist plot of the devices measured under dark conditions.

We evaluated the effect of polymer on the performance of photovoltaic devices with an inverted structure of indium tin oxide (ITO)/nickel oxide (NiO_x_)/(2‐(3,6‐Dimethyl‐9H‐carbazol‐9‐yl)ethyl)phosphonic acid (Me‐2PACz)/perovskite/(6,6)‐phenyl‐C61‐butyric acid methyl ester (PCBM)/2,9‐dimethyl‐4,7‐diphenyl‐1,10‐phenanthroline (BCP)/Ag (Figure 5A). In Figure 5B, the control device exhibits the highest PCE of 24.8%, with a *V*_OC_ of 1.15V, *J*_SC_ of 26.1 mA cm^−2^, and FF of 82.3%. Through the polymer‐assisted optimization, the optoelectronic performance of the devices is significantly improved, with a champion PCE of 26.4%, *V*_OC_ of 1.17 V, *J*_SC_ of 26.2 mA cm^−2^, and FF of 86.0%. The integrated photocurrent (25.1 mA cm^−2^) from the external quantum efficiency (EQE) data is consistent with the *J*_SC_ value (Figure S34). The steady‐state power outputs of the devices stabilize at the maximum power point (MPP) (control: 24.2% and target: 26.0%) under one sun illumination for over 600 s, as shown in Figure 5C. The statistics of *J*–*V* parameters for 20 devices with and without the polymer modification were recorded (inset of Figure 5C; Figure S35), demonstrating the good reproducibility of the performance improvement.

Notably, the FF of the PSC modified by the polar polymer is one of the highest values reported for perovskite photovoltaics with polymer‐modified active layers reported in the recent literature (Figure 5D; Table S3). In prior studies, polymers have been utilized as an effective additive to regulate the properties of the perovskite layers, but the impact of the polymer polarity on the solvent properties and the solvent‐incorporated intermediate phase has not been elucidated. The obvious increased *V*_OC_ and FF upon polymer incorporation can be attributed to the improved perovskite film morphology with enhanced crystallinity, suppressed trap states, and improved homogeneity. This is further supported by the minimal hysteresis observed in the target sample (Figure S36). Figure 5E shows the MPP tracking of PCE for the control and target encapsulated devices in a continuous light‐soaking test. The polymer‐engineered device retained 92% of the initial PCE after aging for 1000 h. In contrast, the control device degraded to 53% of the initial value within 950 h. The improved stability comes from the significantly improved crystallinity of the perovskite films and suppressed traps as delineated above. In addition, the hydrophobic characteristics of the polymer with the fluorinated groups may also play a beneficial role in reducing the interaction of perovskite surfaces with moisture, as evidenced by the significant enhancement of contact angle in the target film (Figure S37).

To further understand the performance enhancement, the light intensity‐dependent *V*_OC_ was obtained to explore the non‐radiative recombination with and without the polymer incorporation (Figure 5F). The target device exhibits a shallower slope in the fitted line (1.12 k_B_T/q) compared to the control device (1.51 k_B_T/q), where k_B_ is the Boltzmann constant, T is the temperature, and q is the elementary charge. A slope closer to 1 suggests a reduction in trap‐assisted non‐radiative recombination in the target device [54]. This is further confirmed by a lower leakage current density obtained from the dark *J*–*V* plots in Figure 5G. The carrier transport kinetics were assessed by impedance spectra deduced from Nyquist plots of devices under dark conditions (Figure 5H). Compared with the control device, the target devices show augmented recombination resistance, from 10 100 to 24 550 Ω, and suppressed series resistance, from 25 to 20 Ω (Figure S38 and Table S4), indicating improved charge transport and reduced non‐radiative recombination. This accounts for the high FF observed in the target devices. This finding implies that the polar polymer can effectively suppress the trap density and help improve film quality.

## 3 Conclusion

In this study, we introduced a solvent engineering strategy, leveraging the highly polar units of fluorinated polymers to stabilize the intermediate phase and enable the fabrication of efficient inverted PSCs. The enhanced interaction between the polar polymer and the solvent molecules regulates solvent diffusion and evaporation, effectively regulating the dynamic evolution of different phases in the solvent and enabling the controlled release and reaction of precursor ions. Subsequently, the gradual transformation process provides advantages for tailoring the crystallization dynamics to a significant degree, leading to the formation of high‐quality perovskite films with higher crystallinity and fewer defects compared to the control samples. As a result, we achieved a PCE of 26.4% for PSCs with an FF of 86.0%. Moreover, the optimized device demonstrated good stability, retaining 92% of its initial efficiency after 1000 h of continuous light soaking under the maximum power point operation. The polar‐polymer‐enabled strategy reported herein can be generalized to other perovskite compositions and solvent systems or scenarios where significant modifications of solvent properties are needed. The high tunability of solvent properties is expected to benefit large‐area devices and even modules where slot die and other solution processing methods are employed. Overall, this work provides a new polarity‐regulated strategy for manipulating the solvent properties and the crystallization dynamics of perovskite films, which holds immense potential for the optimization of a wide range of solvent‐processed device technologies.

## Conflicts of Interest

The authors declare no conflicts of interest.

## Supporting information




**Supporting File**: adma72141‐sup‐0001‐SuppMat.docx.

## Data Availability

The data that support the findings of this study are available in the supplementary material of this article.
