# Supplementary material for: Polarize the Solvent to Regulate the Intermediate Phase and Dynamic Crystallization of Perovskite Films
Source: Adv Mater. 2026 Jan 13;38(12):e19793. doi: 10.1002/adma.202519793 (PMC12933009; doi:10.1002/adma.202519793)
Supplement: Supplementary file 1 — Supporting File: adma72141‐sup‐0001‐SuppMat.docx. [file ADMA-38-e19793-s001.docx]

Supporting Information

Polarize the Solvent to Regulate the Intermediate Phase and Dynamic Crystallization of Perovskite Films

Zhuoqiong Zhang, Yunfan Wang, Weizhen Wang, Yulan Huang, Shanchao Ouyang, Yonggui Sun, Fei Wang, Xianfang Zhou, Guichuan Xing, Shu Kong So, Guozhong Xing, Hanlin Hu*, Songhua Cai*, Sai-Wing Tsang*, Tom Wu*

**Experimental details**

*Materials*

Cesium iodide (CsI), N,N-dimethylformamide (DMF, anhydrous), dimethyl sulfoxide (DMSO, anhydrous), 2-propanol (IPA, anhydrous), and anisole (AN, anhydrous) were purchased from Sigma-Aldrich. Formamidinium iodide (FAI), methylammonium bromide (MABr), methylammonium chloride (MACl), phenethylammonium iodide (PEAI) and BCP were purchased from Xi’an Yuri Solar Co., Ltd. Lead bromide (PbBr_2_), lead iodide (PbI_2_) and Me-2PACz were purchased from Tokyo Chemical Industry. PS, FPS, PPFS, and PoTFMPA (all molecular weight is ~6 kDa) were purchased from Polymer Source. Inc. NiOx nanoparticles and the patterned ITO were purchased from Advanced Electron Technology Co. Ltd. All the chemical materials were used as received. The polymers were dissolved in the solution at a concentration of 2 mg mL^-1^ to study their impact on the solvent properties.

*Perovskite film preparation and device fabrication*

The patterned ITO substrates were sequentially cleaned with detergent, deionized water, acetone, and IPA. Prior to spin coating, the substrates were treated with UV-Ozone for 30 mins. NiO_x_ (7 mg/ml in deionized water) was then spin-coated onto the ITO substrates at 3000 rpm for 30 s and annealed at 100 °C for 10 min in ambient conditions. The samples were subsequently transferred to a nitrogen-filled glovebox, where Me-2PACz (0.5 mg/ml in IPA) was spin-coated. Then CsI (19.5 mg), FAI (232.8 mg), PbI_2_ (721.7 mg), PbBr_2_ (28.5 mg), MACl (18 mg), and MABr (7.9 mg) were dissolved in a solvent mixture of DMF/DMSO (v:v, 4:1) for the perovskite precursor solution. The prepared precursor was spin-coated: 1000 rpm for 5 s followed by 5000 rpm for 40 s. During the second step, 200 µL of AN was dripped onto the sample right before the spin-coating ended. The polymer was introduced by dissolving it in the AN at a concentration of 0.5 mg mL^-1^. The coated substrates were immediately annealed at 100 °C for 30 min. After that, the films underwent post-treatment via spin-coating with PEAI, followed by subsequent spin-coating of PCBM and BCP. Finally, the films were transferred to a thermal evaporator, where a 100 nm Ag layer was deposited to complete the device fabrication.

*Characterizations*

FTIR spectroscopy was performed using a PerkinElmer FTIR Spectrometer. TGA was conducted using a Netzsch TG 209 F3 instrument. STEM imaging was performed using an aberration-corrected STEM microscopy (Spectra300, Thermoﬁsher, USA) equipped with a ﬁeld-emission gun and operated at an accelerating voltage of 300 kV. Its samples were prepared by directly dipping a drop of MAI-PbI_2_-DMSO solution onto copper grids. To minimize damage to the intermediate phase during high-resolution imaging, the electron probe beam current was reduced to 1 pA. The probe convergence angle was set to 24.5 mrad, and the HAADF detector angular range was 57–200 mrad. STEM images in Figure 2 were processed using ABSF filtering to reduce noise. XRD tests were carried out with a D2 Phaser instrument using Cu Kα radiation (*λ* = 0.154 nm). AFM images of perovskite films were obtained using Bruker Dimension Icon. SEM was performed with Field Emission Scanning Electron Microscope (Tescan MAIA3). PL mapping was collected by WITec alpha300 R Raman System. TRPL was measured by FL980 fluorescence spectrometer (Edinburgh). In-situ optical measurements were carried out inside a nitrogen-filled glovebox (Figure S24). A laser intensity was maintained at 8 mW cm^-2^ for the PL test. The same setup was used for in-situ absorption measurements by replacing the laser light with a halogen and LED lamp. PDS was performed using a 1 kW Xe arc lamp and a ¼ m grating monochromator (Oriel). The pump beam was modulated at 13 Hz with a mechanical chopper. Samples, immersed in perfluorohexane as the deflection fluid, were irradiated by a parallel probe beam (He–Ne laser, Uniphase, Model 1103P). Deflection signals from the probe beam were captured with a TEMic position sensor and processed using a Stanford Research SR830 lock-in amplifier. TRPL spectra were acquired using a Hamamatsu streak camera system with a temporal resolution of 1 ps. TA spectra were measured using an Ultrafast System HELIOS TA spectrometer. The excitation source was a Coherent Astrella-1K-F Ti: Sapphire Amplifier operating at 1 kHz with a pulse duration of less than 100 fs. A 600 nm pump pulse (0.16 µJ cm^-2^) was generated using a Light Conversion TOPAS-C optical parametric amplifier. *J*-*V*, SCLC, and *V*_OC_-light dependent measurements were performed using a Keithley 2400 source meter unit under AM 1.5G illustration at 100 mW cm^-2^ with a standard silicon solar cell (certified by NREL) as the reference. The unencapsulated devices were tested with a delay time of 100 ms. The effective device area (0.0468 cm²) was calibrated using a shadow mask during the measurements. During the MPP test, the devices were encapsulated using Loctite AA 3493 light-cure adhesive. MPP tracking was performed under a 1-sun white LED array in ambient air. The EQE spectrum was measured by a QER3-011 solar cell spectral response measurement system (Enli Technology Co. Ltd., Taiwan).

*Theoretical Calculation*

The quantum chemistry calculation was applied to study the dipole moment of different polymers as well as the interaction energy between them and DMSO by using the ORCA 5.0.4 package.^[1]^ The structure of each polymer was simplified by one repeating unit with an end group saturated by hydrogen atoms. The conformational searching method was firstly applied in order to determine the most probable dimer configuration of polymer and DMSO with Molclus and xtb program.^[2,3]^ The geometry optimization of the all-polymer: DMSO dimer after conformational searching was achieved using the B97-3c method. The dimer interaction energy was carried out based on the optimized dimer geometry under the level of ωB97M-V/def2-QZVP. A quadruple-zeta basis set was selected to minimize the basis set superposition error (BSSE) issue. The geometry of individual polymers and DMSO was optimized with the B97-3c method as well. The dipole moment of each individual molecule was obtained under the level of ωB97M-V/def2-TZVP. The input file for calculation and visualization was achieved with the assistance of Multiwfn program.^[4,5]^

**
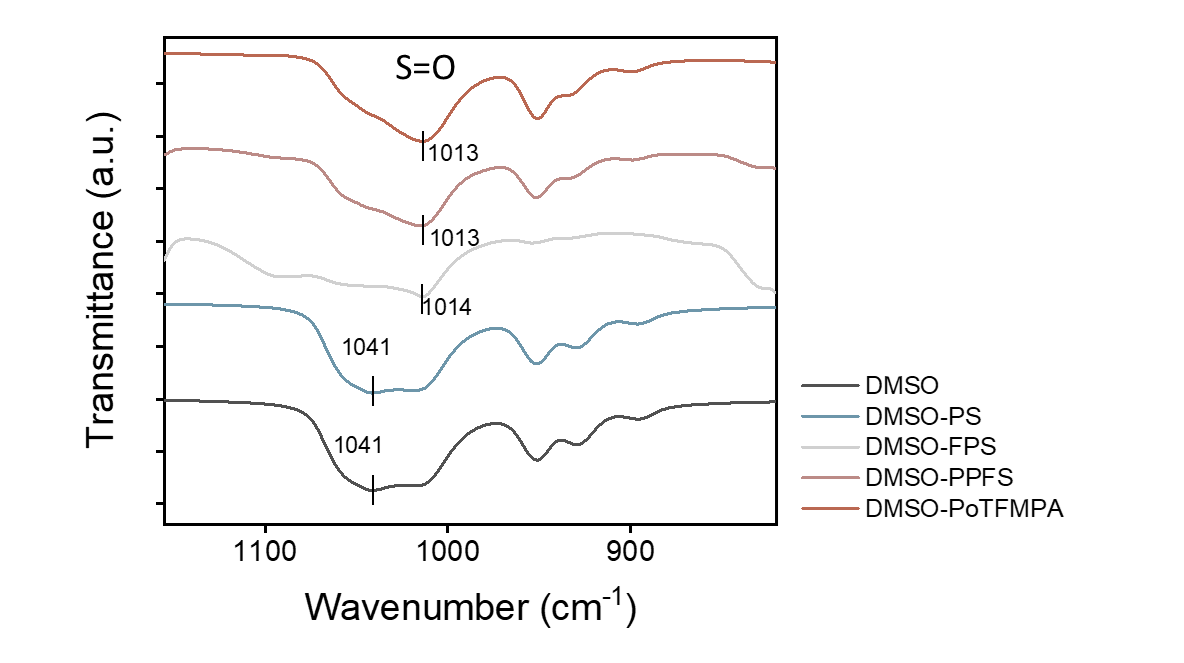
**

**Figure S1.** Interactions between DMSO and polymers. FTIR spectroscopy of pure DMSO and the polymer in DMSO solution.


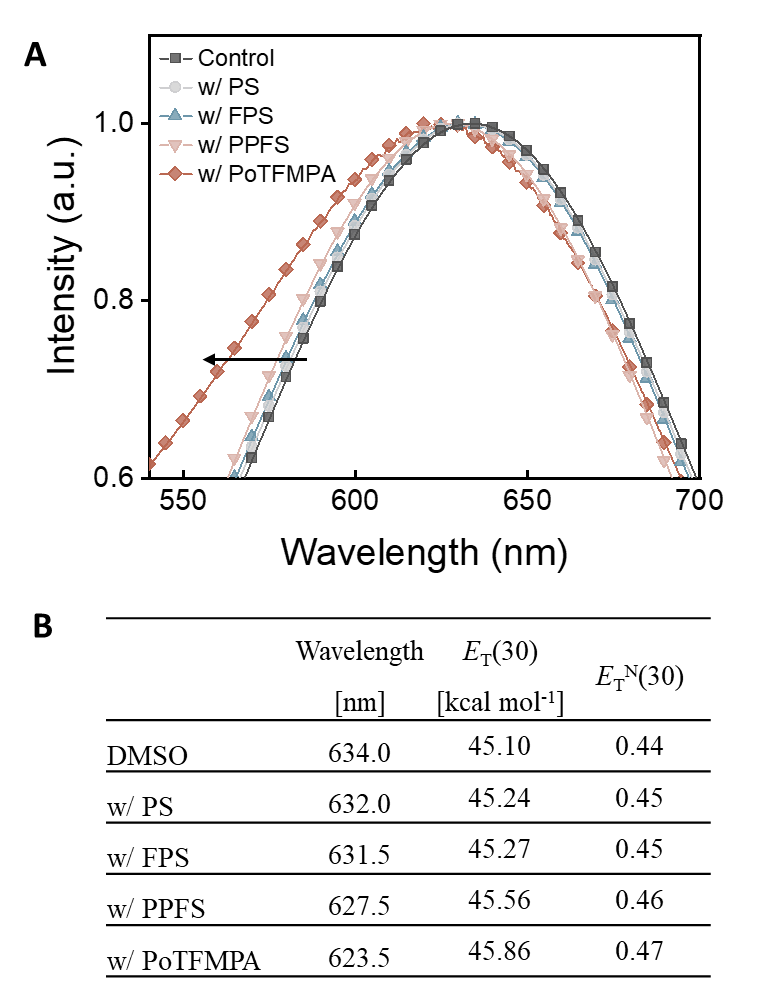


**Figure S2.** (A) UV-vis spectra of betaine dye in pure DMSO and in the polymer-DMSO solution. (B) Summary of solvent polarity values.

Note: *E*_T_(30) can be calculated based on the maximum absorption of the betaine dye according to the following equation:^[6]^

*E*_T_(30)/(kcal mol^-1^) = $hc{ṽ}_{\max}N_{A}=28591/\lambda_{\max}$

where *h* is the Planck’s constant, *c* is the speed of light, $N_{A}$ is Avogadro’s number, and ${ṽ}_{\max}$ and $\lambda_{\max}$ are the wavenumber and wavelength of the maximum absorption band, respectively. To standardize this scale, Reichardt et al. (1983) introduced the dimensionless normalized polar value, $E_{T}^{N},$defined as:^[7]^

$$E_{T}^{N}=\frac{E_{T}\left( \mathrm{solvent} \right)-E_{T}(\mathrm{TMS})}{E_{T}\left( \mathrm{water} \right)-E_{T}(\mathrm{TMS})}= \frac{E_{T}\left( \mathrm{solvent} \right)-30.7}{32.4}$$

This scale utilizes water and tetramethylsilane (TMS) as the most polar and nonpolar reference solvents, respectively.


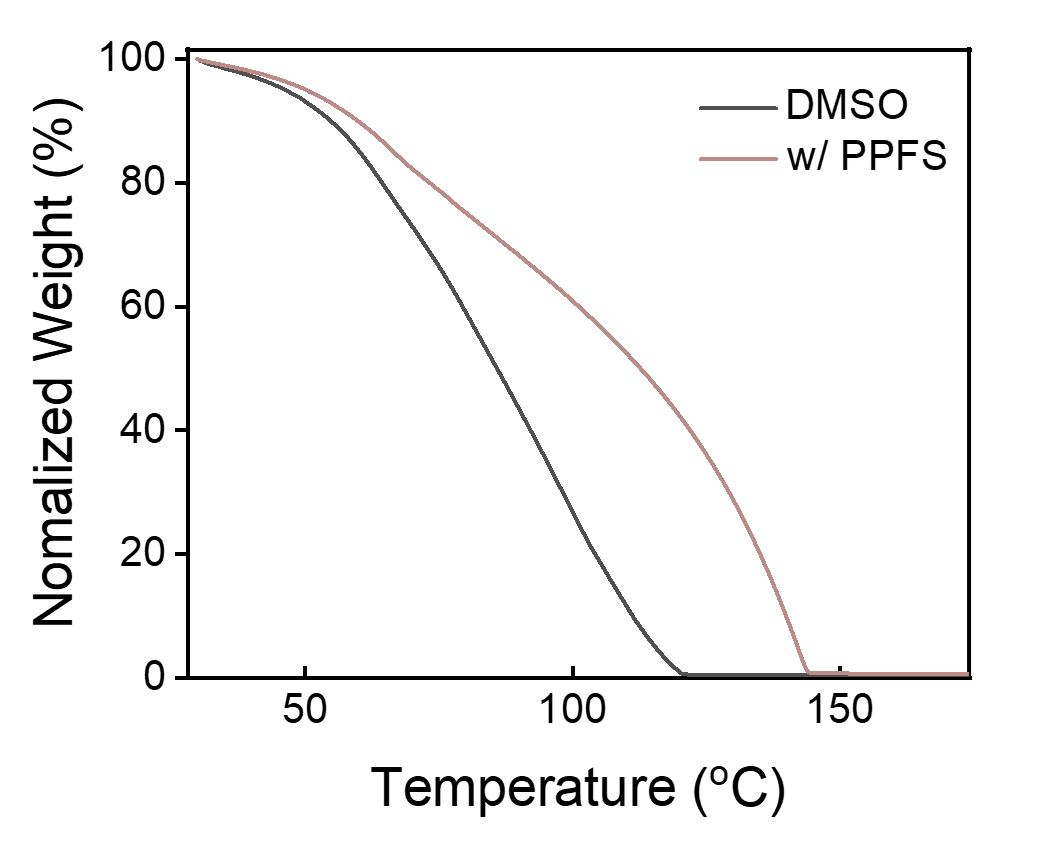


**Figure S3.** TGA curves of pure DMSO without and with the PPFS polymer additive.


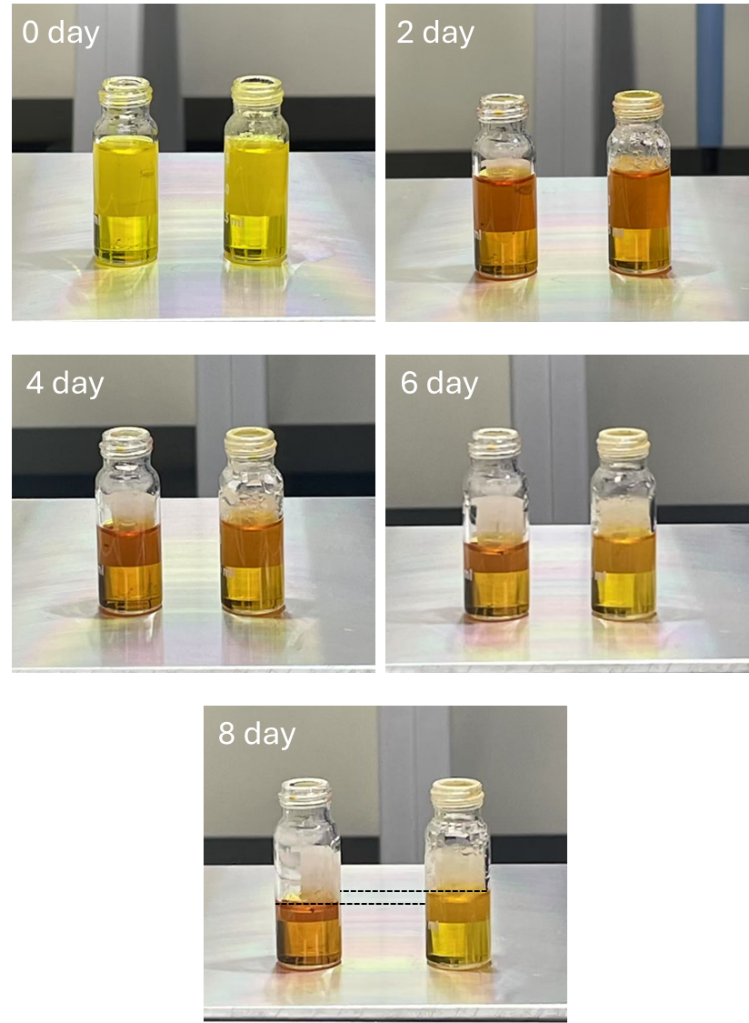


**Figure S4.** Photographs of the perovskite solution with antisolvent, without (left) and with (right) PPFS polymer, heated at 100 °C. The solution without polymer evaporates during heating, while the polymer-containing solution shows reduced evaporation.

**
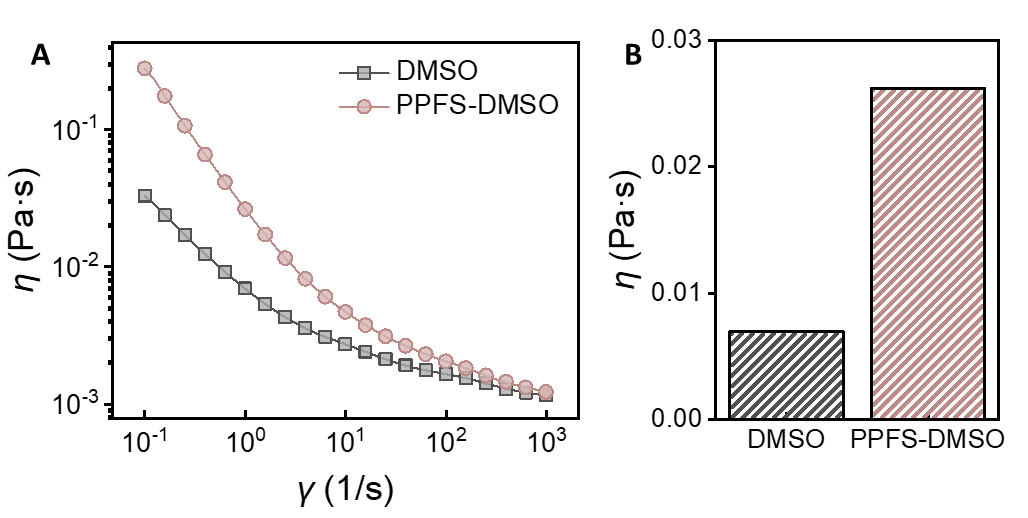
**

**Figure S5.** (A) Viscosity of pure DMSO and PPFS-incorporated DMSO measured across various shear rates. (B) Apparent solution viscosity at a shear rate of 1 s^−1^.

**
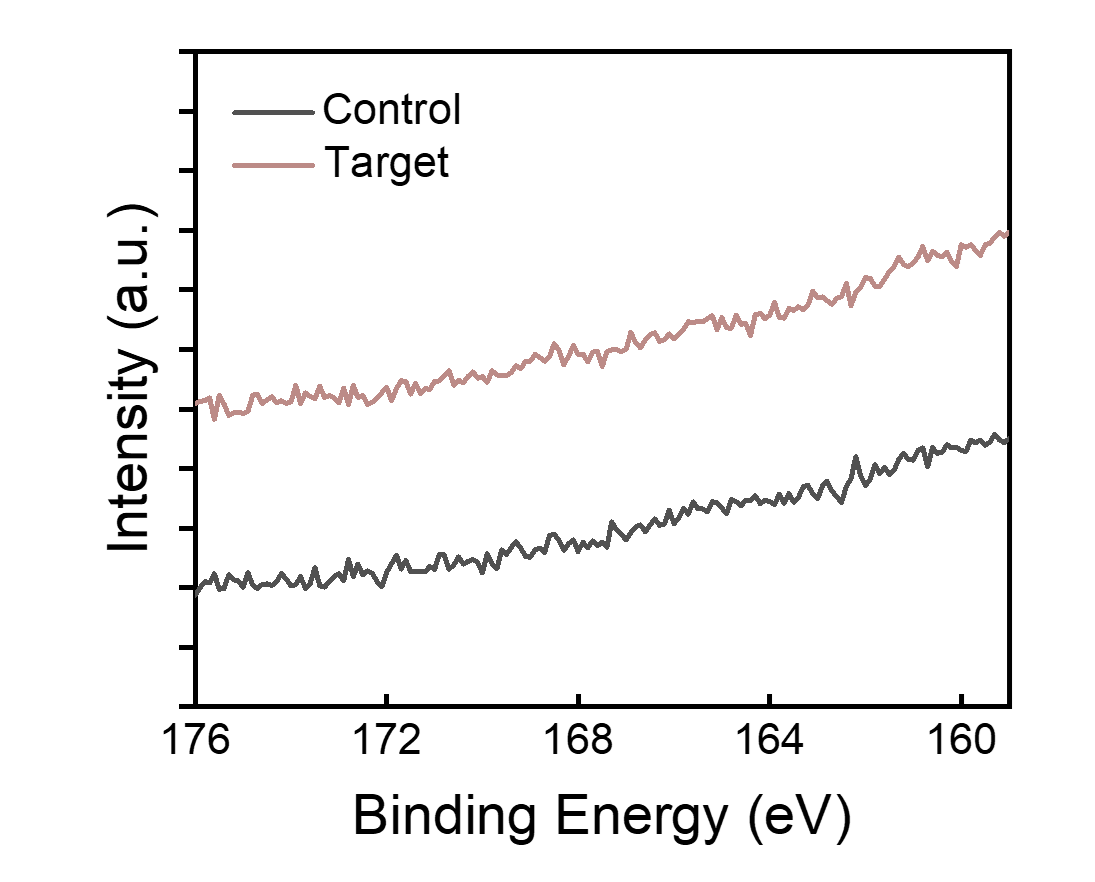
**

**Figure S6.** High-resolution S 2p X-ray photoelectron spectroscopy of control and polymer-incorporated perovskite films.

**
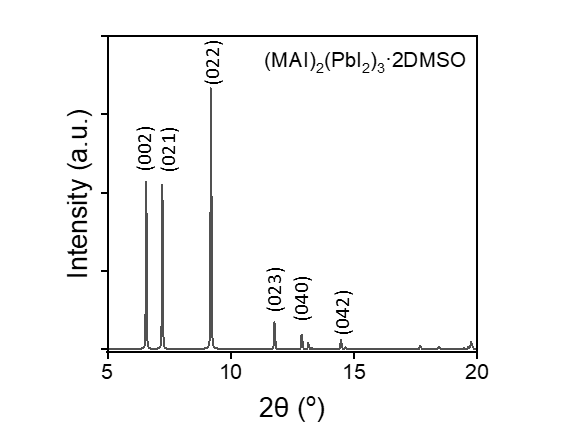
**

**Figure S7.** Simulated powder XRD patterns of (MAI)_2_(PbI_2_)_3_·2DMSO single crystal.^[8]^

**
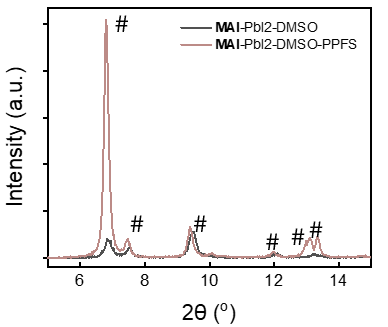
**

**Figure S8.** X-ray diffraction (XRD) patterns of films without and with the addition of PPFS. “#” indicates MAI-PbI_2_-DMSO phase.

**
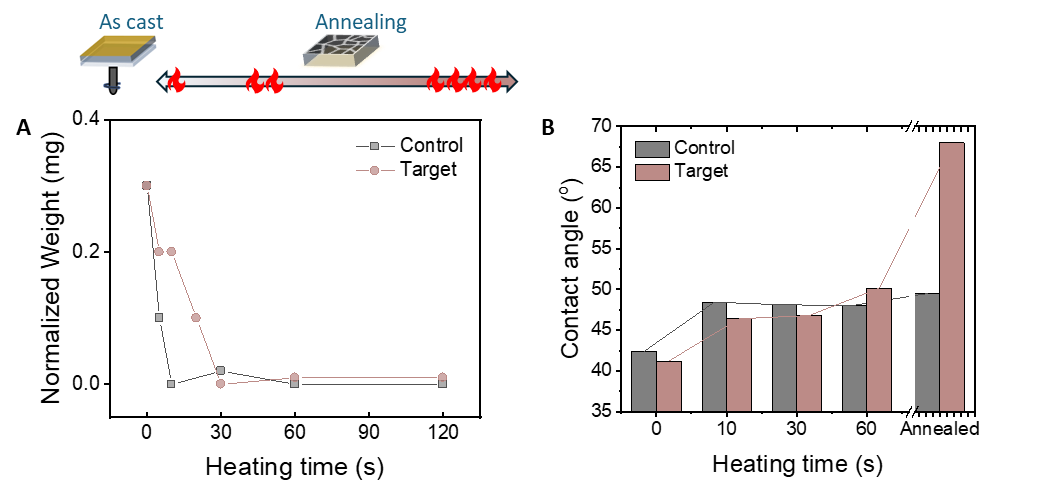
**

**Figure S9.** Detection of residual solvent in wet films with (A) weight loss measurements and (B) contact angle measurements. The “0 s” corresponds to the as-cast wet film, followed by heating the sample at 100 °C for varying durations. The target sample requires a longer time to stabilize compared to the control.

**
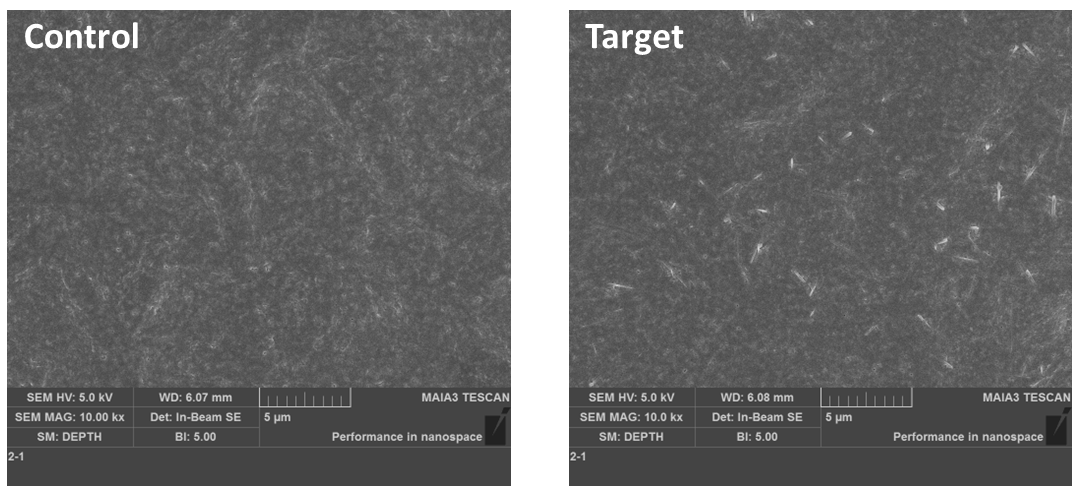
**

**Figure S10.** SEM images of perovskite wet films without and with polymer treatment.

**
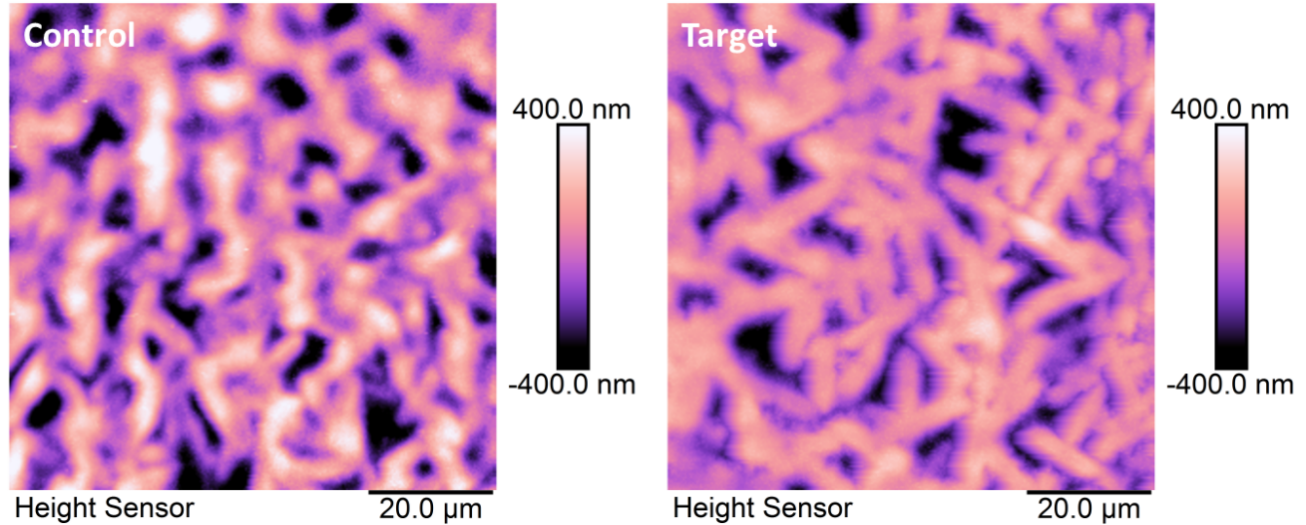
**

**Figure S11.** AFM images of perovskite wet films without and with polymer treatment.

**
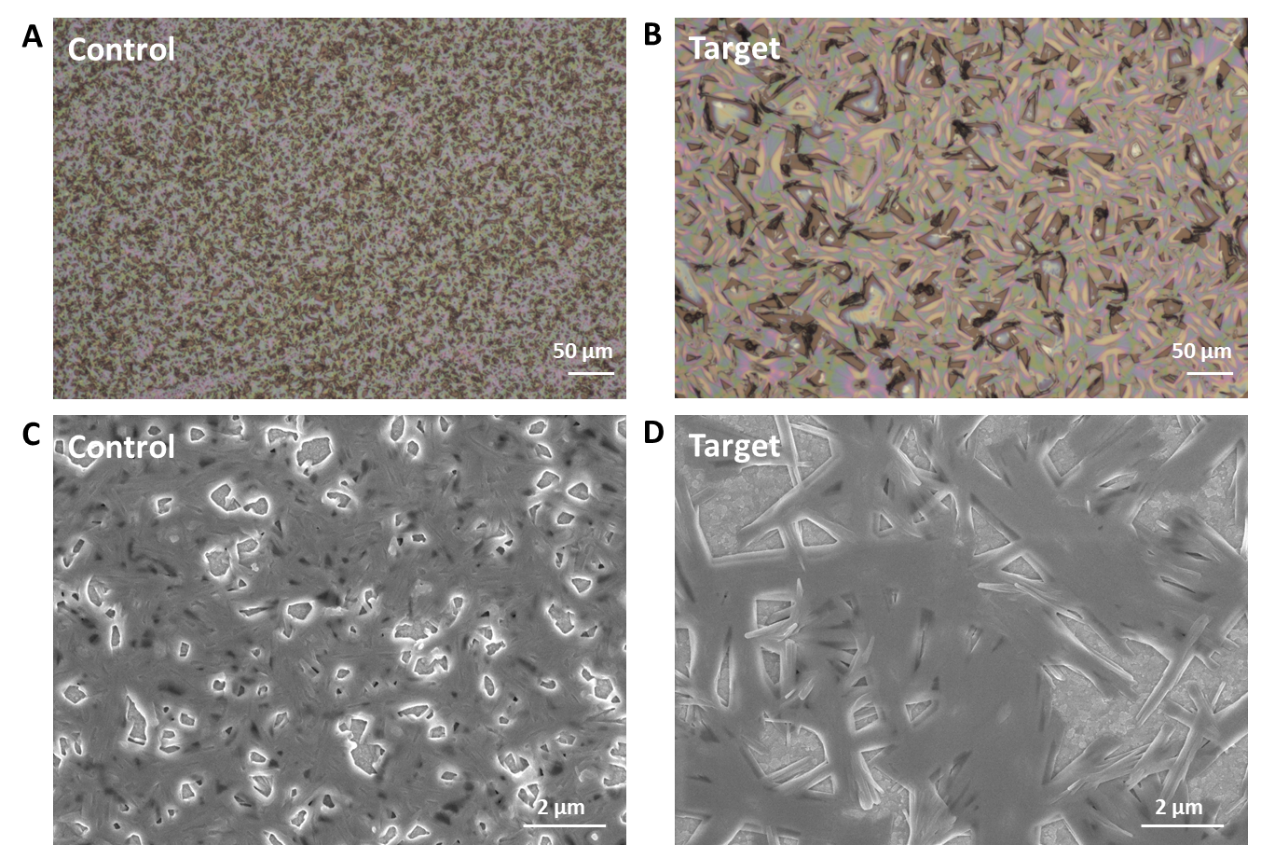
**

**Figure S12.** Optical images of spin-coated MAI-PbI_2_-DMSO films (A) without and (B) with polymer treatment.


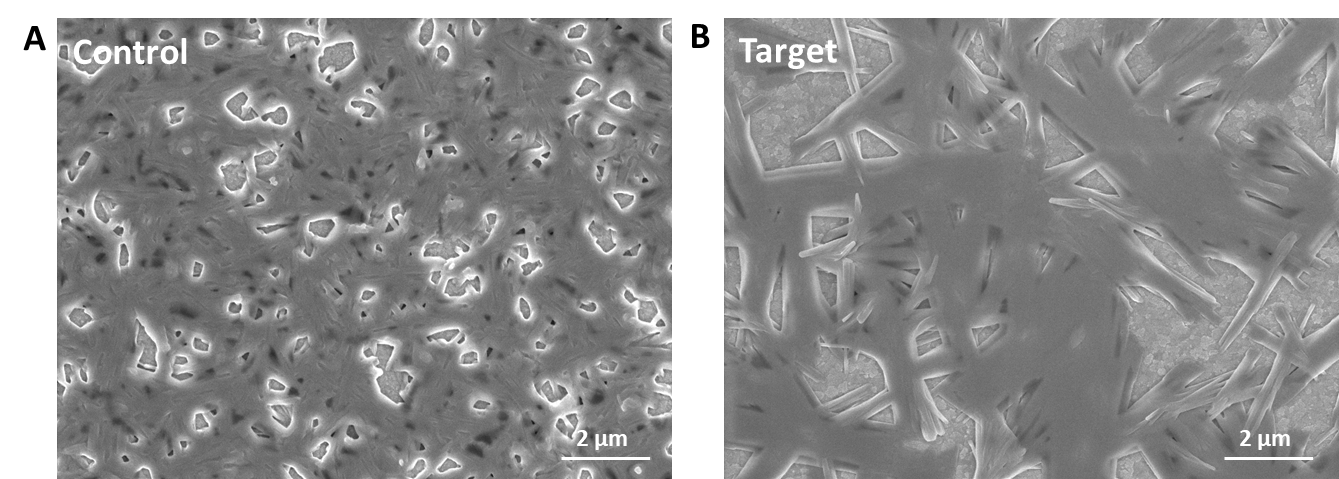


**Figure S13.** SEM images of spin-coated MAI-PbI_2_-DMSO films (A) without and (B) with polymer treatment.


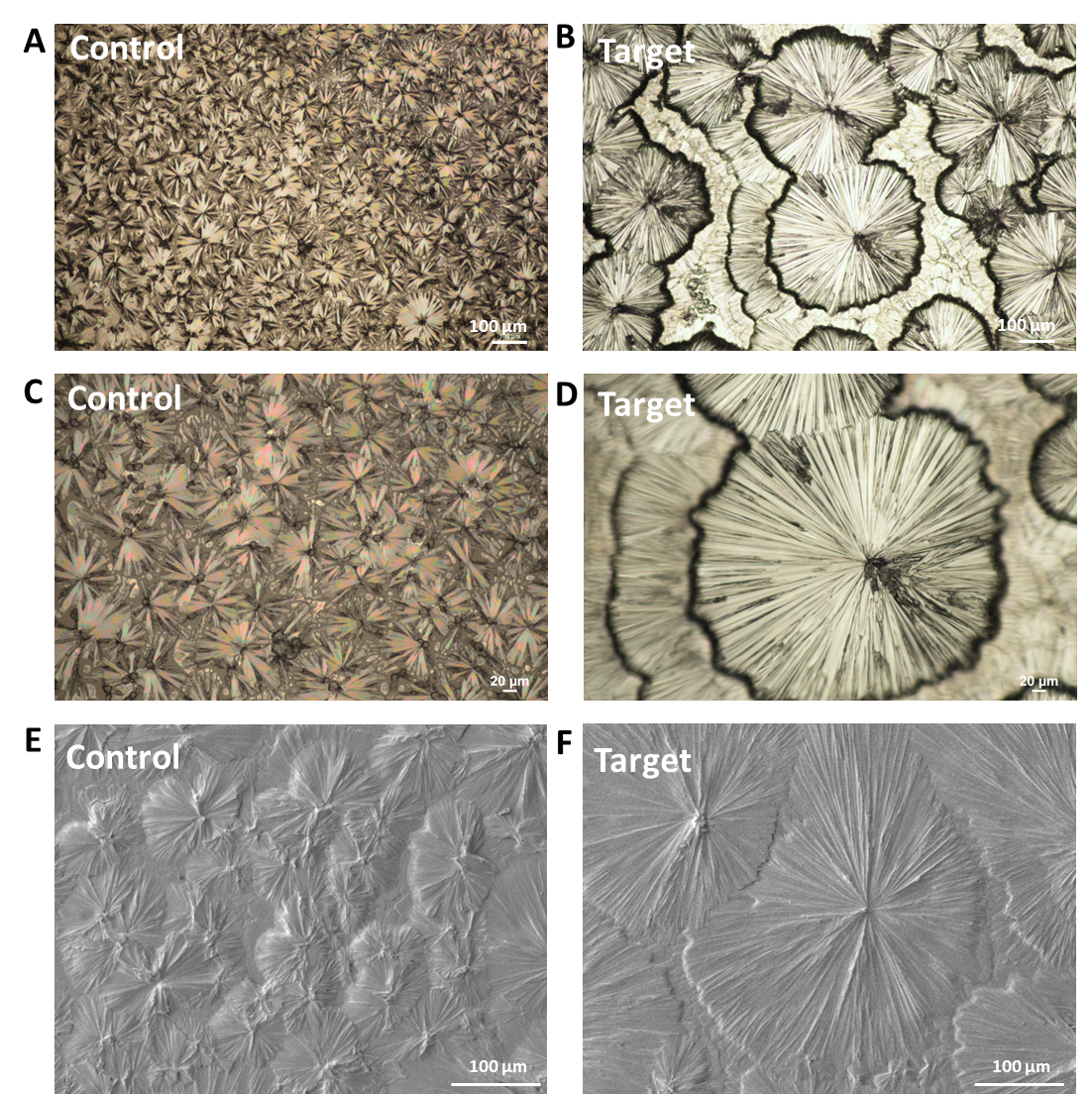


**Figure S14.** Optical images of drop-coated MAI-PbI_2_-DMSO films (A, C) without and (B, D) with polymer treatment at different scales.


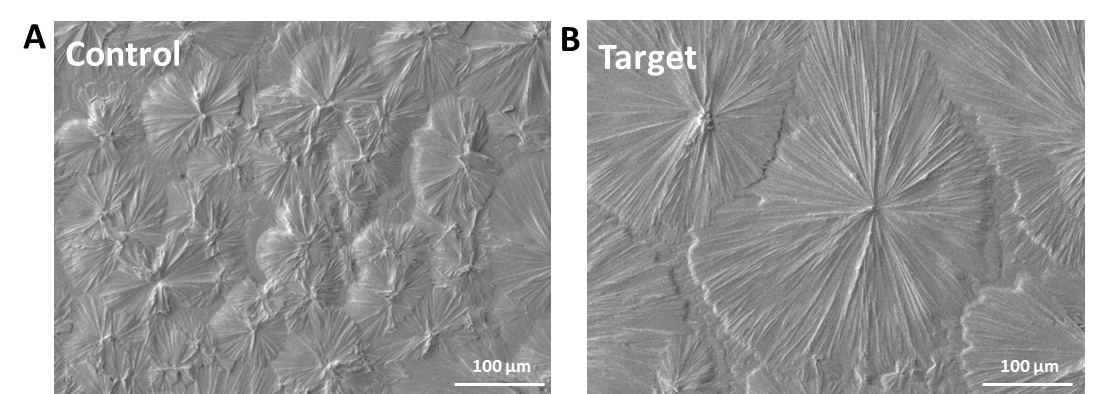


**Figure S15.** SEM images of drop-coated MAI-PbI_2_-DMSO films (A) without and (B) with polymer treatment.

**
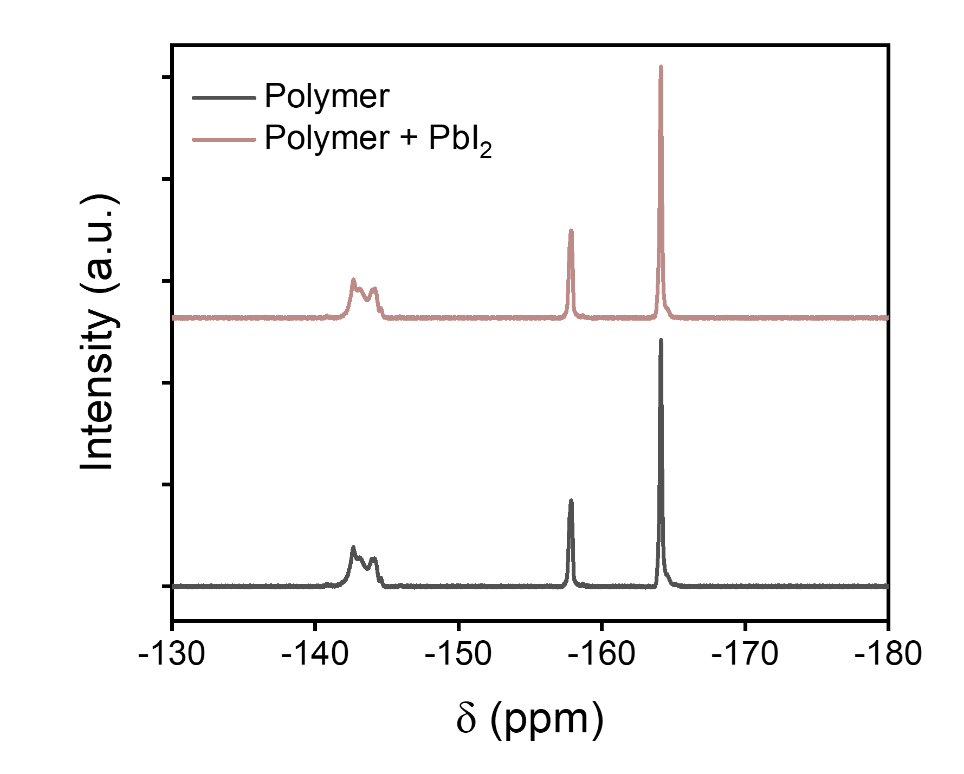
**

**Figure S16.** ^19^F-Nuclear Magnetic Resonance Spectroscopy spectrum of the pure polymer and the polymer mixed with PbI_2_.

**
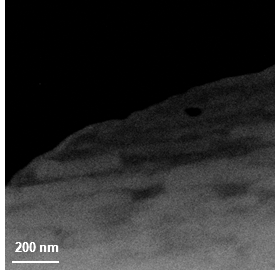
**

**Figure S17.** Low-magnification HAADF-STEM image of control sample.


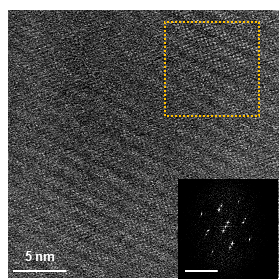


**Figure S18.** High-resolution HAADF-STEM image of the control sample with the corresponding Fast Fourier transform pattern (inset, scale bar 5 nm^-1^).


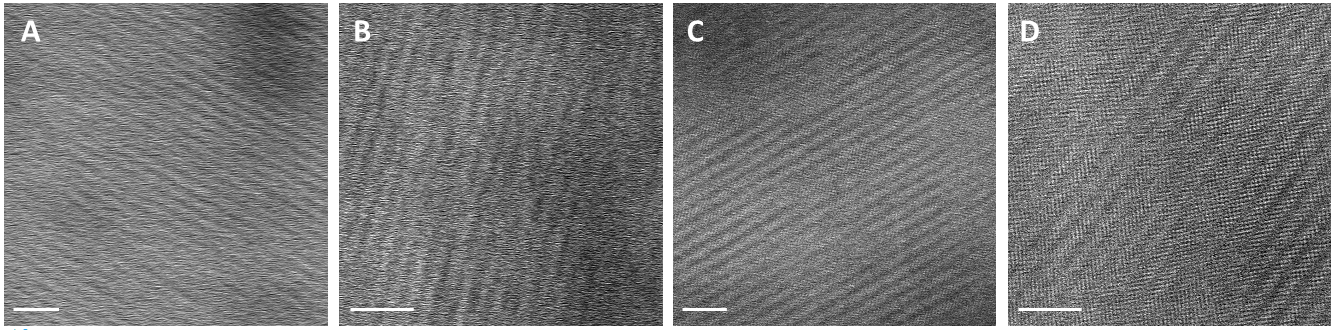


**Figure S19**. HAADF-STEM images taken from different samples (A, B) without and (C, D) with polymer treatment, confirming spatial homogeneity. Scale bar: 5 nm. Note that the control samples exhibited poorer crystallization compared with the polymer-treated samples.

**
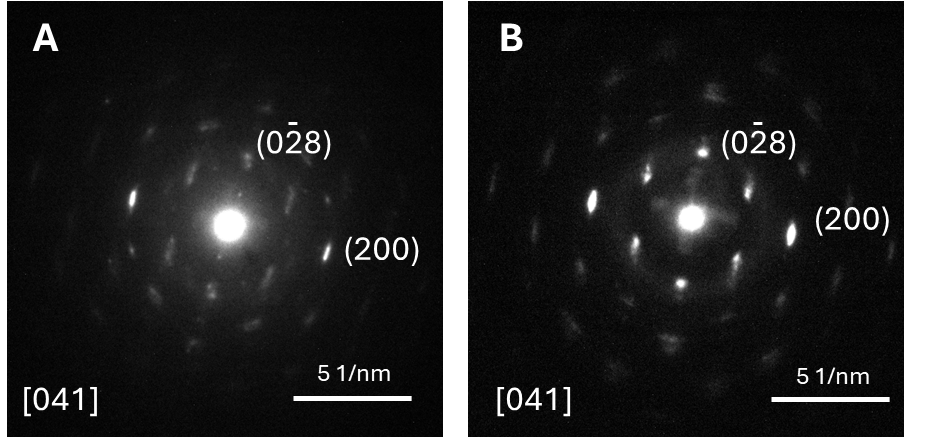
**

**Figure S20.** The selected area electron diffraction patterns of MAI-PbI_2_-DMSO films (A) without and (B) with polymer treatment.

**
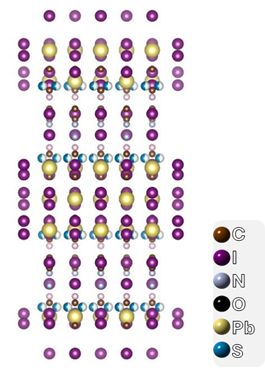
**

**Figure S21.** Atomic model of MA_2_Pb_3_I_8_·2DMSO viewed from [010] direction.^[8]^


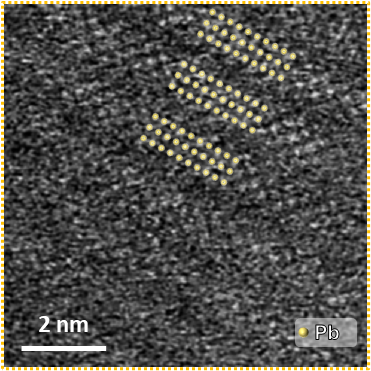


**Figure S22.** Enlarged view of the yellow square region in Figure S18, indicating a similar periodicity compared with the atomic model shown in Figure S21.


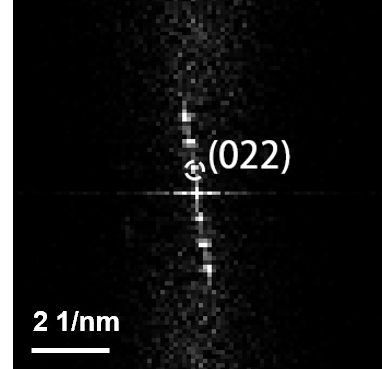


**Figure S23.** The Fast Fourier transform pattern corresponding to the lattice parameter of 9.6 Å in **Figure 2E**.

**
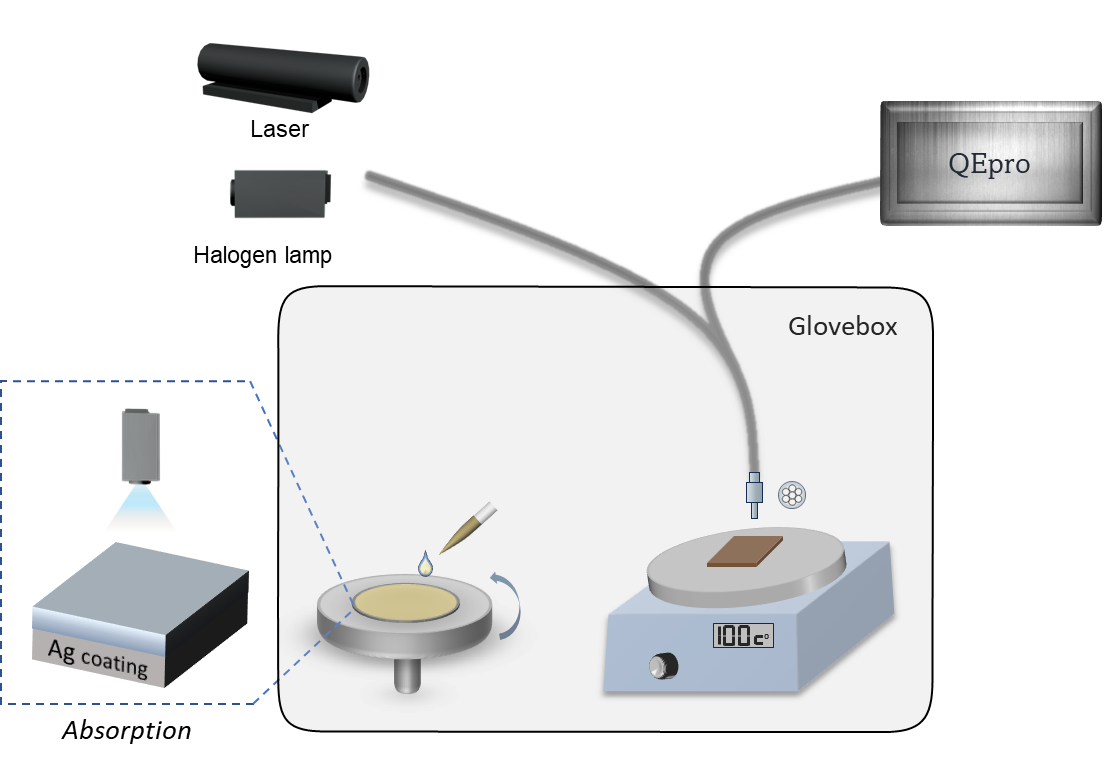
**

**Figure S24.** Schematic diagram of in-situ optical setup.


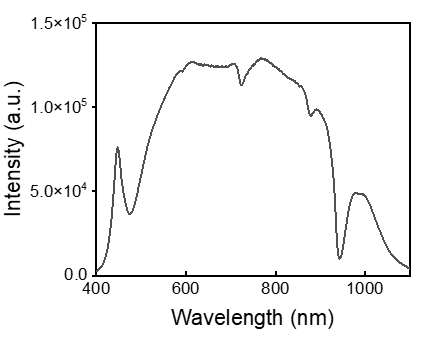


**Figure S25**. The spectrum of the light source for the in-situ absorption test.


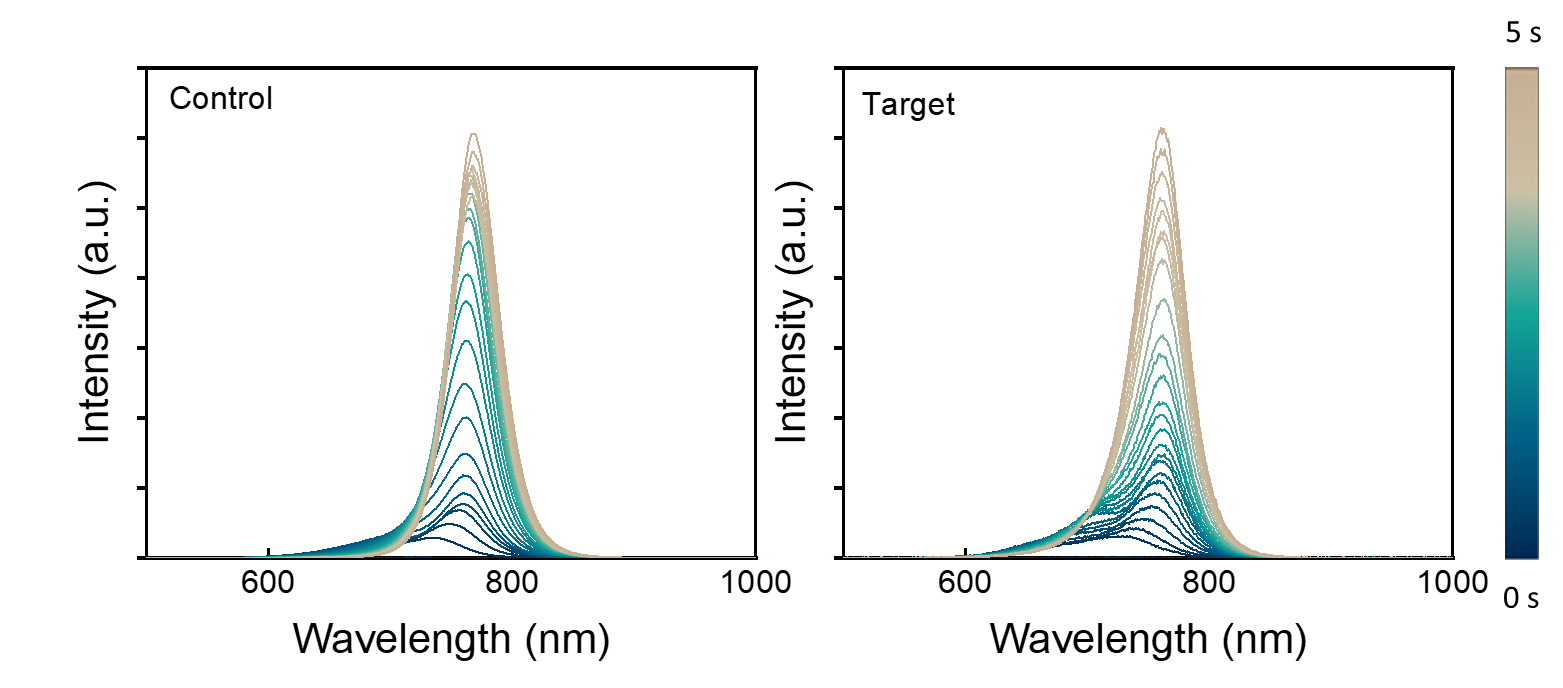


**Figure S26.** In-situ PL evolution during the initial spin-coating process (0–5 s) for films without and with polymer treatment.

**
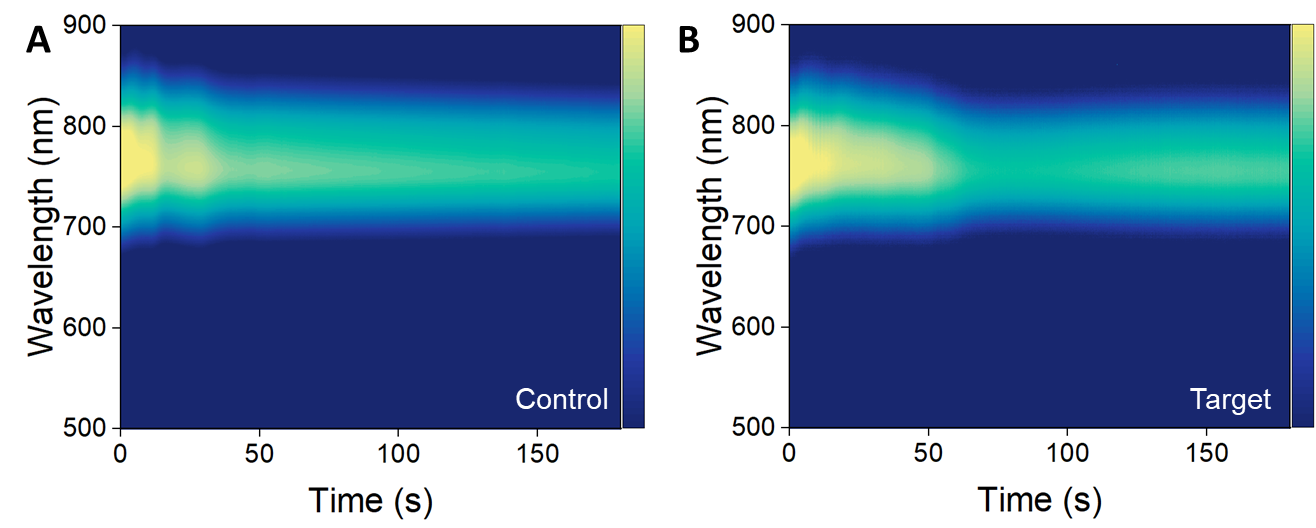
**

**Figure S27.** 2D contour maps of PL spectra for perovskite films (A) without and (B) with polymer treatment during the annealing process.

**
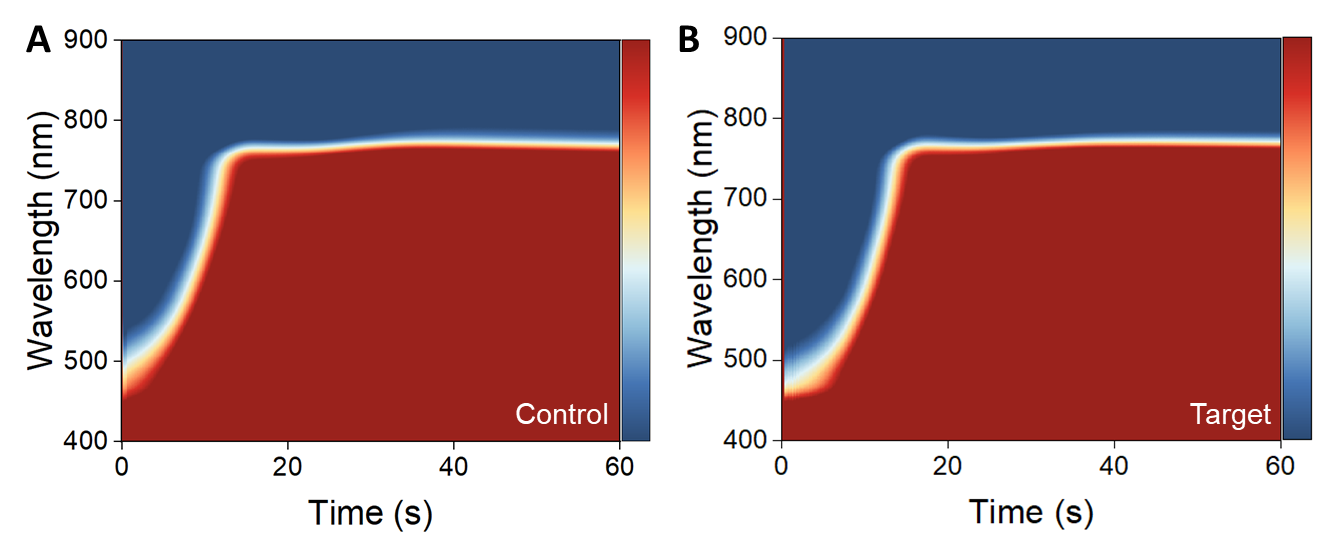
**

**Figure S28.** 2D contour maps of the UV–Vis spectra for perovskite films (A) without and (B) with polymer treatment during the annealing process.

**
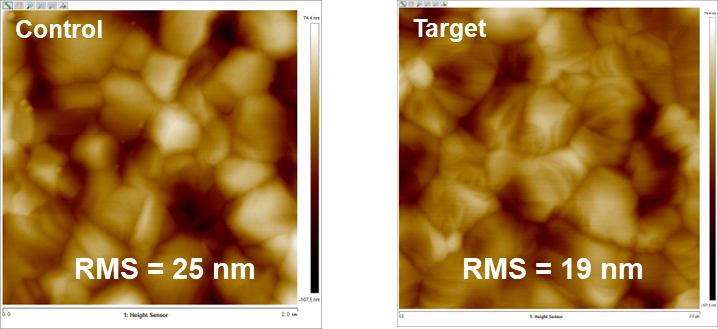
**

**Figure S29.** AFM height images of perovskite films without and with polymer treatment.

**
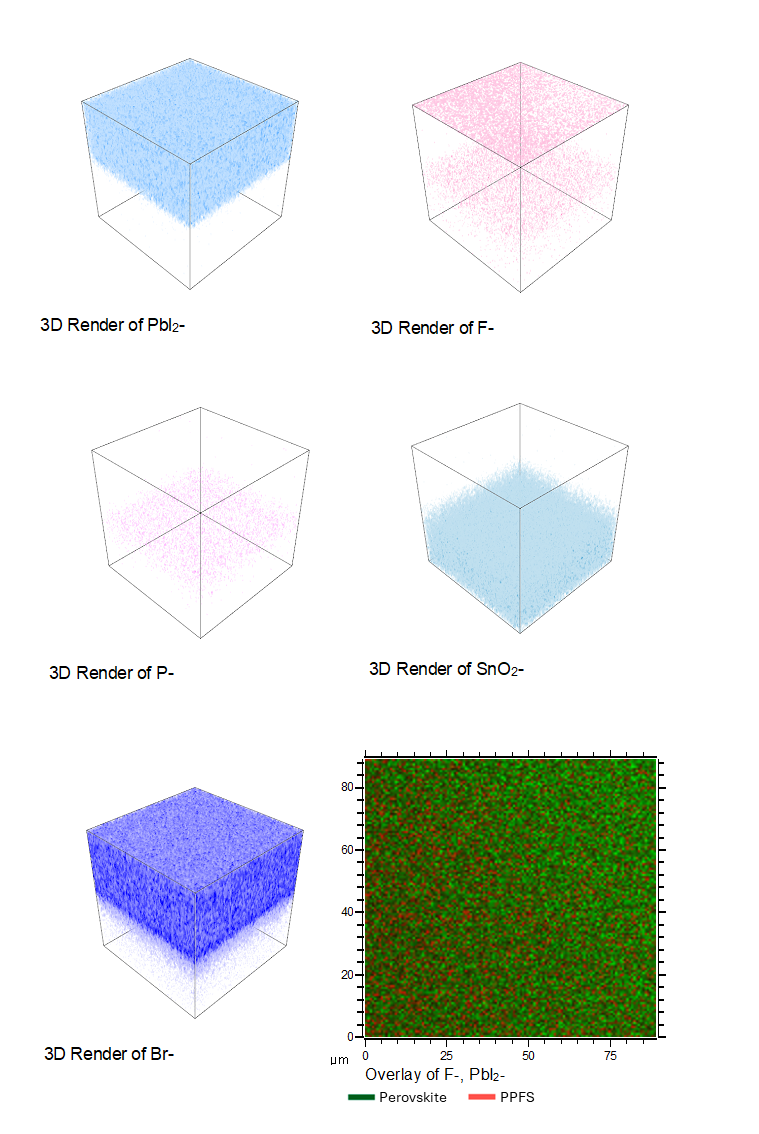
**

**Figure S30.** Time-of-flight-SIMS 3D topographies showing the distributions of PbI_2_^-^, F^-^, P^-^, SnO_2_^-^, and Br^-^, along with corresponding 2D topographies.


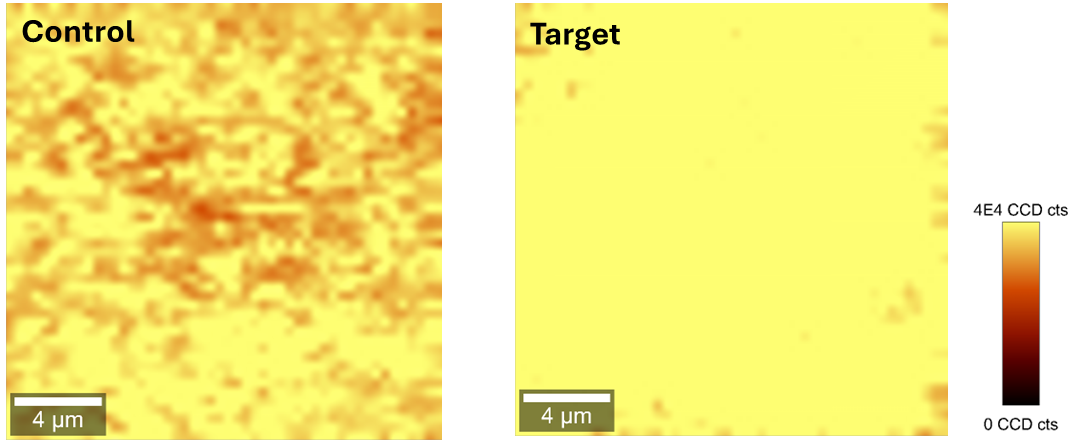


**Figure S31.** PL mappings of perovskite films without and with polymer treatment.


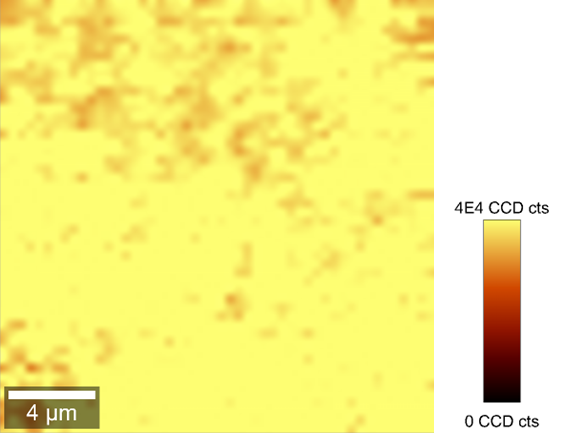


**Figure S32.** PL mappings of perovskite film treated with a higher polymer concentration (1 mg mL^-1^).

**
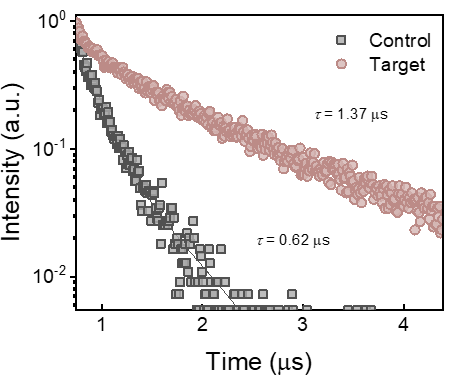
**

**Figure S33.** TRPL of perovskite films without and with polymer treatment.


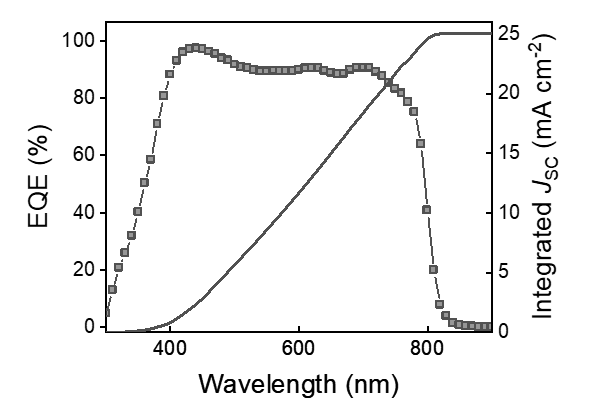


**Figure S34.** EQE spectrum of the target device.


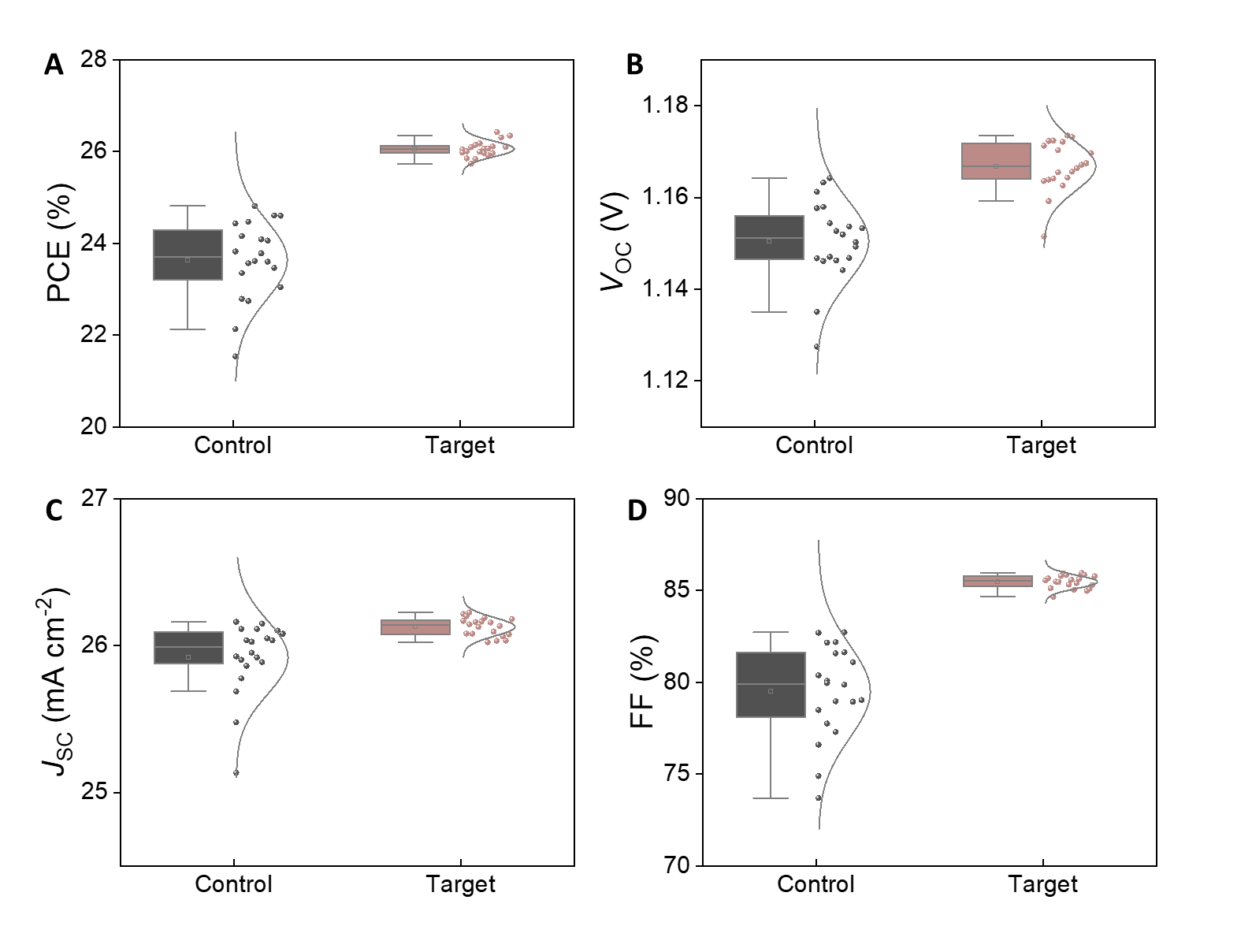


**Figure S35.** Statistical analysis of the photovoltaic performance of control and target PSCs based on data from 20 individual devices.


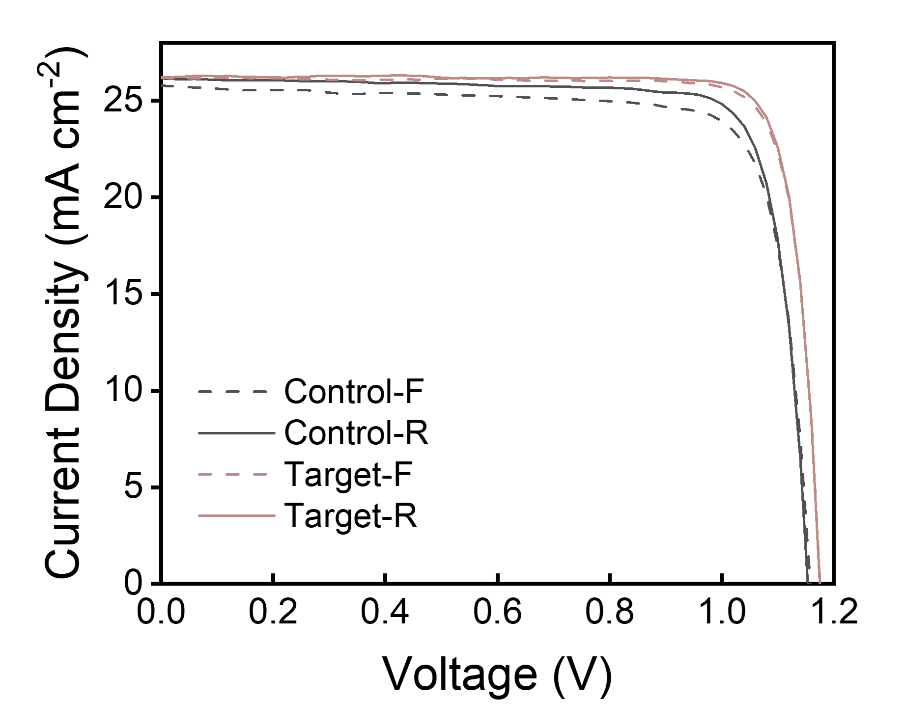


**Figure S36.** *J*–*V* curves of control and target PSCs in the forward and reverse scans.

*
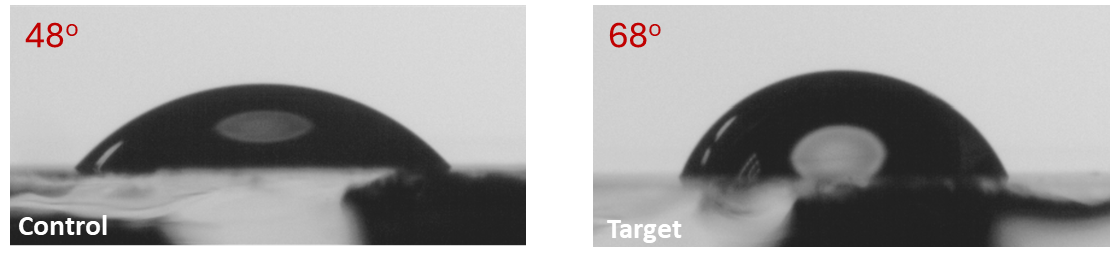
*

**Figure S37.** Contact angles of perovskite films without and with polymer treatment.

*
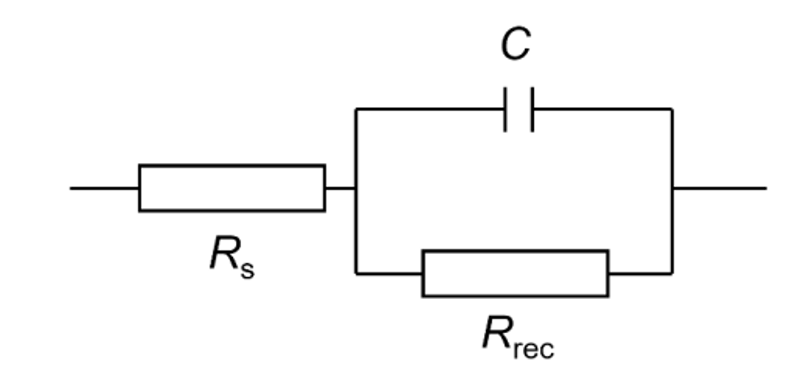
*

**Figure S38.** Equivalent-circuit model used for RIS fitting of PSCs, consisting of the series resistance (*R*_s_), the recombination resistance (*R*_rec_) and the capacitance (*C*).

**Table S1**. Trap density value extracted from SCLC tests.

| Sample | | *V*_TFL_ (V) | *N*_t_ (10^15^ cm^-3^) |
| --- | --- | --- | --- |
| Electron | Control | 0.13 | 0.83 |
|  | Target | 0.08 | 0.51 |
| Hole | Control | 1.06 | 6.75 |
|  | Target | 0.43 | 2.74 |

**Table S2**. Summary of TA parameters obtained from the three-exponential fitting equation.^[9]^

| Sample | A_1_ | *τ*_1_ (ps) | A_2_ | *τ*_2_ (ps) | A_3_ | *τ*_3_ (ps) |
| --- | --- | --- | --- | --- | --- | --- |
| Control | 1.24 | 4.0 | 2.76 | 148 | 3.58 | 1500 |
| Target | 19.16 | 4.2 | 12.03 | 353 | 8.31 | 2730 |

**Table S3.** Comparison of FF and PCE for the previous studies using polymer modification of the active layer.

| Polymer | *V*_OC_  (V) | *J*_SC_  (mA cm^-2^) | FF  (%) | PCE  (%) | Reference |
| --- | --- | --- | --- | --- | --- |
| FTPA | 1.161 | 24.25 | 82.41 | 23.22 | ^[10]^ |
| *𝛽*-pV2F | 1.177 | 24.80 | 84.30 | 24.60 | ^[11]^ |
| S-polymer | 1.110 | 25.57 | 83.17 | 23.52 | ^[12]^ |
| PDMS | 1.168 | 25.67 | 81.4 | 24.41 | ^[13]^ |
| TA-NI | 1.18 | 25.10 | 81.0 | 23.87 | ^[14]^ |
| Rb-PAA | 1.179 | 25.49 | 82.20 | 24.69 | ^[15]^ |
| [Se-MI][BF4] | 1.165 | 26.04 | 82.66 | 25.10 | ^[16]^ |
| PAA | 1.160 | 25.20 | 82.46 | 24.19 | ^[17]^ |
| POF-HDDA | 1.201 | 25.72 | 80.13 | 24.76 | ^[18]^ |
| PEGDAM | 1.153 | 25.04 | 82.91 | 23.94 | ^[19]^ |
| ABC | 1.199 | 25.18 | 81 | 24.45 | ^[20]^ |
| PAH | 1.192 | 25.49 | 82.72 | 25.14 | ^[21]^ |
| AETA-BCF | 1.192 | 25.71 | 84.1 | 25.76 | ^[22]^ |
| PVBN | 1.175 | 25.31 | 85.1 | 25.30 | ^[23]^ |
| PECL | 1.15 | 23.98 | 83.79 | 23.11 | ^[24]^ |
| PEIE | 1.17 | 24.95 | 83.31 | 24.32 | ^[25]^ |
| PSMA | 1.15 | 24.37 | 84.0 | 23.57 | ^[26]^ |
| TFEMA | 1.15 | 25.03 | 82.9 | 23.91 | ^[27]^ |
| PTHM | 1.162 | 24.15 | 85.6 | 24.03 | ^[28]^ |
| PAM | 1.211 | 25.63 | 83.88 | 26.05 | ^[29]^ |
| IP1 | 1.16 | 25.74 | 83.98 | 25.06 | ^[30]^ |
| AMPS-DEA | 1.181 | 26.29 | 82.99 | 25.78 | ^[31]^ |
| HPSiM | 1.18 | 25.48 | 84.41 | 25.38 | ^[32]^ |
| SBMA: HEA | 1.194 | 26.12 | 83.85 | 26.15 | ^[33]^ |
| Poly-PAE | 1.197 | 25.92 | 84.45 | 26.20 | ^[34]^ |
| CDVFB | 1.186 | 26.02 | 84.42 | 26.05 | ^[35]^ |

**Table S4**. Fitting parameters of PSCs from Nyquist plots.

|  | *R*_s_  (Ω) | *R*_rec_  (Ω) |
| --- | --- | --- |
| Control | 25 | 10100 |
| Target | 20 | 24550 |

Reference

[1] F. Neese, *WIREs Comput. Mol. Sci.* **2012**, *2*, 73.

[2] L. Tian, “Molclus program, Version 1.12,” can be found under http://www.keinsci.com/research/molclus.html (accessed January 5, 2025)**.**

[3] C. Bannwarth, S. Ehlert, S. Grimme, *J. Chem. Theory Comput.* **2019**, *15*, 1652.

[4] T. Lu, F. Chen, *J. Comput. Chem.* **2012**, *33*, 580.

[5] T. Lu, *J. Chem. Phys.* **2024**, *161*, 082503.

[6] R. Pandian, H. Burda, I. Alfurayj, C. Reichardt, C. Burda, *J. Phys. Chem. B* **2024**, *128*, 6990.

[7] C. Reichardt, E. Harbusch-Görnert, *Liebigs Annalen der Chemie* **1983**, *1983*, 721.

[8] Y. Rong, Z. Tang, Y. Zhao, X. Zhong, S. Venkatesan, H. Graham, M. Patton, Y. Jing, A. M. Guloy, Y. Yao, *Nanoscale* **2015**, *7*, 10595.

[9] H. Zhu, B. Shao, Z. Shen, S. You, J. Yin, N. Wehbe, L. Wang, X. Song, M. Abulikemu, A. Basaheeh, A. Jamal, I. Gereige, M. Freitag, O. F. Mohammed, K. Zhu, O. M. Bakr, *Nat. Photon.* **2025**, *19*, 28.

[10] M. Li, R. Sun, J. Chang, J. Dong, Q. Tian, H. Wang, Z. Li, P. Yang, H. Shi, C. Yang, Z. Wu, R. Li, Y. Yang, A. Wang, S. Zhang, F. Wang, W. Huang, T. Qin, *Nat. Commun.* **2023**, *14*, 573.

[11] G. Li, Z. Su, L. Canil, D. Hughes, M. H. Aldamasy, J. Dagar, S. Trofimov, L. Wang, W. Zuo, J. J. Jerónimo-Rendon, M. M. Byranvand, C. Wang, R. Zhu, Z. Zhang, F. Yang, G. Nasti, B. Naydenov, W. C. Tsoi, Z. Li, X. Gao, Z. Wang, Y. Jia, E. Unger, M. Saliba, M. Li, A. Abate, *Science* **2023**, *379*, 399.

[12] K. Kim, J. Han, S. Lee, S. Kim, J.-M. Choi, J.-S. Nam, D. Kim, I. Chung, T.-D. Kim, S. Manzhos, S. J. Choi, J. W. Song, D. S. Kim, J. Y. Do, I. Jeon, *Adv. Energy Mater.* **2023**, *13*, 2203742.

[13] T. Wang, Z. Wan, X. Min, R. Chen, Y. Li, J. Yang, X. Pu, H. Chen, X. He, Q. Cao, G. Feng, X. Chen, Z. Ma, L. Jiang, Z. Liu, Z. Li, W. Chen, X. Li, *Adv. Energy Mater.* **2024**, *14*, 2302552.

[14] Z. Chen, Q. Cheng, H. Chen, Y. Wu, J. Ding, X. Wu, H. Yang, H. Liu, W. Chen, X. Tang, X. Lu, Y. Li, Y. Li, *Adv. Mater.* **2023**, *35*, 2300513.

[15] C. Zhao, H. Zhang, M. Almalki, J. Xu, A. Krishna, F. T. Eickemeyer, J. Gao, Y. M. Wu, S. M. Zakeeruddin, J. Chu, J. Yao, M. Grätzel, *Adv. Mater.* **2023**, *35*, 2211619.

[16] Y. Shen, G. Xu, J. Li, X. Lin, F. Yang, H. Yang, W. Chen, Y. Wu, X. Wu, Q. Cheng, J. Zhu, Y. Li, Y. Li, *Angew. Chem.* **2023**, *62*, e202300690.

[17] S. Lin, S. Wu, D. Guo, H. Huang, X. Zhou, D. Zhang, K. Zhou, W. Zhang, Y. Hu, Y. Gao, C. Zhou, *Small Methods* **2023**, *7*, 2201663.

[18] J. Zhang, Z. Li, F. Guo, H. Jiang, W. Yan, C. Peng, R. Liu, L. Wang, H. Gao, S. Pang, Z. Zhou, *Angew. Chem.* **2023**, *62*, e202305221.

[19] L. Li, Z. Huang, X. Meng, Z. Xing, B. Fan, J. Li, Y. Chen, *Adv. Mater.* **2024**, *36*, 2310752.

[20] H. Guo, G. W. Yoon, Z. J. Li, Y. Yun, S. Lee, Y.-H. Seo, N. J. Jeon, G. S. Han, H. S. Jung, *Adv. Energy Mater.* **2024**, *14*, 2302743.

[21] S. Cao, T. Zheng, Z. Bi, B. Z. Taye, S. Luo, H. A. Tauqeer, Y. Zheng, Y. Zhuo, Z. Liang, H. Wen, H. Wu, K. Wang, D. Yang, S. (Frank) Liu, H. Wang, H.-Y. Hsu, X. Xu, *Small* **2025**, *21*, 2410716.

[22] B. Zhang, H. Zeng, H. Yin, D. Zheng, Z. Wan, C. Jia, T. Stuyver, J. Luo, T. Pauporté, *Energy Environ. Sci.* **2024**, *17*, 5532.

[23] Z. Dai, Y. Yang, X. Huang, S. Wan, L. Yuan, H. Wei, S. Nie, Z. Liu, Y. Wu, R. Chen, H. Wang, *Nano Energy* **2024**, *131*, 110190.

[24] T. Wang, Y. Li, Q. Cao, J. Yang, B. Yang, X. Pu, Y. Zhang, J. Zhao, Y. Zhang, H. Chen, A. Hagfeldt, X. Li, *Energy Environ. Sci.* **2022**, *15*, 4414.

[25] Z. Zhu, K. Mao, K. Zhang, W. Peng, J. Zhang, H. Meng, S. Cheng, T. Li, H. Lin, Q. Chen, X. Wu, J. Xu, *Joule* **2022**, *6*, 2849.

[26] D. Li, Y. Huang, R. Ma, H. Liu, Q. Liang, Y. Han, Z. Ren, K. Liu, P. W.-K. Fong, Z. Zhang, Q. Lian, X. Lu, C. Cheng, G. Li, *Adv. Energy Mater.* **2023**, *13*, 2204247.

[27] G. Yuan, W. Xie, Q. Song, S. Ma, Y. Ma, C. Shi, M. Xiao, F. Pei, X. Niu, Y. Zhang, J. Dou, C. Zhu, Y. Bai, Y. Wu, H. Wang, Q. Fan, Q. Chen, *Adv. Mater.* **2023**, *35*, 2211257.

[28] X. Pu, J. Zhao, Y. Li, Y. Zhang, H.-L. Loi, T. Wang, H. Chen, X. He, J. Yang, X. Ma, X. Li, Q. Cao, *Nano Energy* **2023**, *112*, 108506.

[29] B. Zhang, Q. Zhao, K. Gao, X. Zhang, C. Gao, X. Sun, H. Ji, X. Feng, Y. Han, X. Yan, X. Wang, Z. Shao, S. Pang, K. Chen, G. Cui, *Adv. Sci.* **2025**, *12*, 2503417.

[30] S. Lian, M. Liu, H. Zhu, Y. Wu, A. Dolgormaa, Y. Zhang, H. Zhan, J. Liu, L. Wang, C. Qin, *J. Phys. Chem. Lett.* **2025**, *16*, 4835.

[31] Z. Li, C. Jia, H. Wu, Y. Tang, J. Zhao, Z. Su, X. Gao, S. Qiu, H. Yuan, M. Li, *Angew. Chem.* **2025**, *64*, e202421063.

[32] G. Feng, T. Wang, X. He, H. Chen, W. Lu, Z. Zhou, Q. Cao, X. Li, *Nano Energy* **2025**, *141*, 111084.

[33] C. Shao, J. Ma, G. Niu, Z. Nie, Y. Zhao, F. Wang, J. Wang, *Adv. Mater.* **2025**, *37*, 2417150.

[34] J. Ma, S. Fan, C. Shao, L. Wang, Y. Dong, G. Niu, Z. Nie, S. Yang, J. Wang, H. Yang, *Angew. Chem.* **2025**, *137*, e202425578.

[35] J. Huang, X. Li, Z. Zhang, T. Sun, H. Dong, H. Yu, X. Ma, W. Yang, L. Dai, L. Wang, B. Hu, Y. Shen, M. K. Nazeeruddin, M. Wang, *Adv. Mater.* **2025**, e11684.
